# Comparative analysis of single-stranded DNA donors to generate conditional null mouse alleles

**DOI:** 10.1186/s12915-018-0529-0

**Published:** 2018-06-21

**Authors:** Denise G. Lanza, Angelina Gaspero, Isabel Lorenzo, Lan Liao, Ping Zheng, Ying Wang, Yu Deng, Chonghui Cheng, Chuansheng Zhang, John R. Seavitt, Francesco J. DeMayo, Jianming Xu, Mary E. Dickinson, Arthur L. Beaudet, Jason D. Heaney

**Affiliations:** 1 Department of Molecular and Human Genetics, Baylor College of Medicine, One Baylor Plaza, MS BCM225, Houston, TX 77030 USA; 2 Mouse ES Cell Core, Baylor College of Medicine, 1 Baylor Plaza, Houston, TX 77030 USA; 3 Department of Molecular Biology, Baylor College of Medicine, 1 Baylor Plaza, Houston, TX 77030 USA; 4 Genetically Engineered Mouse Core, Baylor College of Medicine, 1 Baylor Plaza, Houston, TX 77030 USA; 5 Lester and Sue Smith Breast Center, Baylor College of Medicine, 1 Baylor Plaza, Houston, TX 77030 USA; 6 Department of Neuroscience, Baylor College of Medicine, 1 Baylor Plaza, Houston, TX 77030 USA; 7 Reproductive and Developmental Biology Laboratory, National Institute of Environmental Health Sciences, Research Triangle Park, Durham, NC 27709 USA; 8 Department of Molecular Physiology and Biophysics, Baylor College of Medicine, 1 Baylor Plaza, Houston, TX 77030 USA

**Keywords:** CRISPR/Cas9, Conditional null allele, High-throughput production, Genome editing, Single-stranded DNA oligonucleotides, Homology directed repair, Mouse models

## Abstract

**Background:**

The International Mouse Phenotyping Consortium is generating null allele mice for every protein-coding gene in the genome and characterizing these mice to identify gene–phenotype associations. While CRISPR/Cas9-mediated null allele production in mice is highly efficient, generation of conditional alleles has proven to be more difficult. To test the feasibility of using CRISPR/Cas9 gene editing to generate conditional knockout mice for this large-scale resource, we employed Cas9-initiated homology-driven repair (HDR) with short and long single stranded oligodeoxynucleotides (ssODNs and lssDNAs).

**Results:**

Using pairs of single guide RNAs and short ssODNs to introduce loxP sites around a critical exon or exons, we obtained putative conditional allele founder mice, harboring both loxP sites, for 23 out of 30 targeted genes. LoxP sites integrated in cis in at least one mouse for 18 of 23 genes. However, loxP sites were mutagenized in 4 of the 18 in *cis* lines. HDR efficiency correlated with Cas9 cutting efficiency but was minimally influenced by ssODN homology arm symmetry. By contrast, using pairs of guides and single lssDNAs to introduce loxP-flanked exons, conditional allele founders were generated for all four genes targeted, although one founder was found to harbor undesired mutations within the lssDNA sequence interval. Importantly, when employing either ssODNs or lssDNAs, random integration events were detected.

**Conclusions:**

Our studies demonstrate that Cas9-mediated HDR with pairs of ssODNs can generate conditional null alleles at many loci, but reveal inefficiencies when applied at scale. In contrast, lssDNAs are amenable to high-throughput production of conditional alleles when they can be employed. Regardless of the single-stranded donor utilized, it is essential to screen for sequence errors at sites of HDR and random insertion of donor sequences into the genome.

**Electronic supplementary material:**

The online version of this article (10.1186/s12915-018-0529-0) contains supplementary material, which is available to authorized users.

## Background

Over the last decade, the ease with which mammalian genomes can be modified in vitro and in vivo has substantially increased with the advent of genome editing technologies. Protein-guided nucleases (e.g., zinc finger nucleases and TALENS) and RNA-guided endonucleases (e.g., CRISPR/Cas9) enable genome editing at specific targeted sites in genomes through the generation of double-strand breaks (DSBs) and their subsequent repair through imprecise non-homologous end joining (NHEJ) or precise homology-directed repair (HDR) with an exogenous DNA donor template [1]. Due to its ability to direct DSBs to precise locations in the genome with high efficiency [2], the ease of reagent design, and the relative low cost of reagent production, the CRISPR/Cas9 system has become the preferred technology for germline genome editing in dozens of mammalian species, including mouse [3–5]. Numerous publications have illustrated the ease of generating novel mouse lines from injections of Cas9 mRNA or protein and synthetic single-guide RNAs (sgRNAs) [6–13] and reported methods to facilitate the production of edited alleles [13–16].

While NHEJ-mediated null allele production in mice is highly efficient [8, 17], conditional allele generation has proven to be more difficult. Adapting traditional plasmid-based methods to target conditional alleles into the genome of mouse embryos has shown to be inefficient when attempted [8]. Furthermore, production of new plasmid donors is time consuming, ordering existing targeting constructs from repositories is expensive, and modification of the existing targeting constructs to harbor shorter homology arms for CRISPR/Cas9-mediated HDR requires specific expertise, and can be time and cost prohibitive [18]. As an alternative, short (≤ 200 bp) single-stranded oligodeoxynucleotide donors (ssODNs) are fast and relatively inexpensive to produce. Generation of conditional null alleles requires a single pair of sgRNAs targeting intronic sequences flanking exon(s) and a pair of loxP-containing ssODNs; this approach requires two DSBs and two independent HDR events to generate a conditional allele. The reported conditional null founder rates per gene attempted has been less than or equal to 20% using pairs of sgRNAs and ssODNs [8, 13, 19–21]. Importantly, each previous study using ssODNs to create conditional null alleles in mice targeted a single gene or a small subset of genes each under different conditions. Long, single-stranded oligodeoxynucleotide donors (lssDNAs) are also relatively easy and cost effective to produce, may be able to target a conditional allele with a single HDR event, and have recently been shown to be highly efficient at generating both conditional and reporter knock-in alleles [14, 22]. Thus, ssODNs and lssDNAs appear to be the most cost effective and efficient methods of conditional allele production in mice. Whether the apparent efficiencies of single-stranded donor DNAs will be maintained when systematically applied at multiple loci and at scale has not been evaluated.

The International Mouse Phenotyping Consortium (IMPC) and its NIH-funded Knockout Mouse Production and Phenotyping (KOMP^2^) component is producing a null allele mouse line for every protein coding gene in the mouse genome, and phenotyping each null allele line to elucidate gene function in human biology and disease [23]. During Phase I, IMPC/KOMP^2^ utilized a library of C57BL/6 N embryonic stem (ES) cells harboring targeted null and sophisticated, flexible ‘knockout first’ alleles to produce chimeric mice for germline transmission [24–27]. Null allele mouse strains were produced and phenotyped, and sperm was cryopreserved for distribution to the scientific community [28]. To date, this effort has resulted in the production, phenotyping, and distribution of thousands of null allele and conditional null allele lines, representing roughly one-third of the mouse genome [29]. However, with clear advantages in the ease of production and lower cost, the majority of IMPC sites have adopted CRISPR/Cas9 genome editing approaches to supply their phenotyping pipelines with mice harboring critical exon deletion (null) alleles [30–33]. While this approach accomplishes the goal of phenotyping null alleles, the approach does not currently permit the generation of conditional null alleles, which would be highly advantageous to investigators that utilize IMPC/KOMP^2^ resources. Thus, it remains a high priority to develop CRISPR/Cas9 genome editing approaches amenable to high-throughput production of mice harboring conditional null alleles to maximize the allele diversity available for distribution to the scientific community.

Here, we report the results of a large-scale pilot study testing the feasibility of using CRISPR/Cas9-mediated HDR with pairs of sgRNAs and ssODNs to produce conditional null allele mice for the high-throughput IMPC/KOMP^2^ production pipelines. Our results demonstrate that CRISPR/Cas9-mediated HDR with pairs of ssODNs can successfully produce conditional null alleles at multiple loci across the genome. However, integration of loxP sites in *trans* and mutagenesis of loxP sequences impedes efficient conditional null allele production when using paired ssODNs. By contrast, our data suggest that CRISPR/Cas9-mediated HDR with pairs of sgRNAs and a single lssDNA harboring short homology arms and a loxP-flanked exon is more efficient than ssODNs at producing conditional null alleles, and is therefore more appropriate for high-throughput mouse production. Importantly, both approaches facilitate the simultaneous production of animals harboring null alleles when Cas9-induced DSBs are resolved by NHEJ rather than HDR. Thus, conditional alleles for the IMPC repository and null alleles for the phenotyping pipeline can be generated in the same microinjection. Together, our results indicate that lssDNAs are likely to be the best, first choice for scaled conditional null allele production, and that ssODNs are a viable secondary option when conditional allele designs are not amenable to the lssDNA approach. Importantly, our results also highlight the need for stringent quality control benchmarks when employing single-stranded oligodeoxynucleotides as donors for conditional allele production. Both ssODNs and lssDNAs can introduce sequence errors during HDR and can randomly integrate into the genome.

## Results

### Conditional allele design and genotyping schemes for CRISPR/Cas9-mediated HDR with ssODNs

To test whether CRISPR/Cas9-mediated HDR with pairs of sgRNAs and ssODNs can be used to efficiently and reliably produce conditional null alleles across the genome, we selected 30 genes to target for CRISPR/Cas9 genome editing. Genes selected for conditional alleles were either investigator-requested or sourced as previous failures of ES cell-based targeting by the IMPC. To capitalize on resources already available from the IMPC [34], we utilized EUCOMM tm1a targeting vector designs [25] already created for each gene to identify the critical exon or exons to flank with loxP sites. The following criteria were used to select critical exons to flank with loxP sites (‘flox’). The 5′-most exon that (1) is larger than 100 bp in size, (2) allows translation to initiate at the native start site, and (3) once removed from the genome by Cre/loxP recombination, induces a reading frameshift, a premature stop codon, and non-sense-mediated decay of all predicted coding transcripts. In some cases, multiple exons were selected for floxing as one single exon did not meet the 100 bp size requirement or induce a frameshift. All genes with MGI allele designs on the IMPC website have an accompanying GenBank file, which clearly annotates the critical exon or exons for targeting, listed as Ensembl exon IDs. Pairs of sgRNAs were identified to generate DSBs within introns 5′ and 3′ to the critical exon(s). Selected target sequences were at least 100 bp 5′ or 3′ from the exon to be floxed (and any neighboring exons, when necessary) to minimize effects on splicing, ideally flanked by sequence without repetitive elements or GC content greater than 80% or less than 20% (Additional file 1: Table S1).

**Fig. 1:**
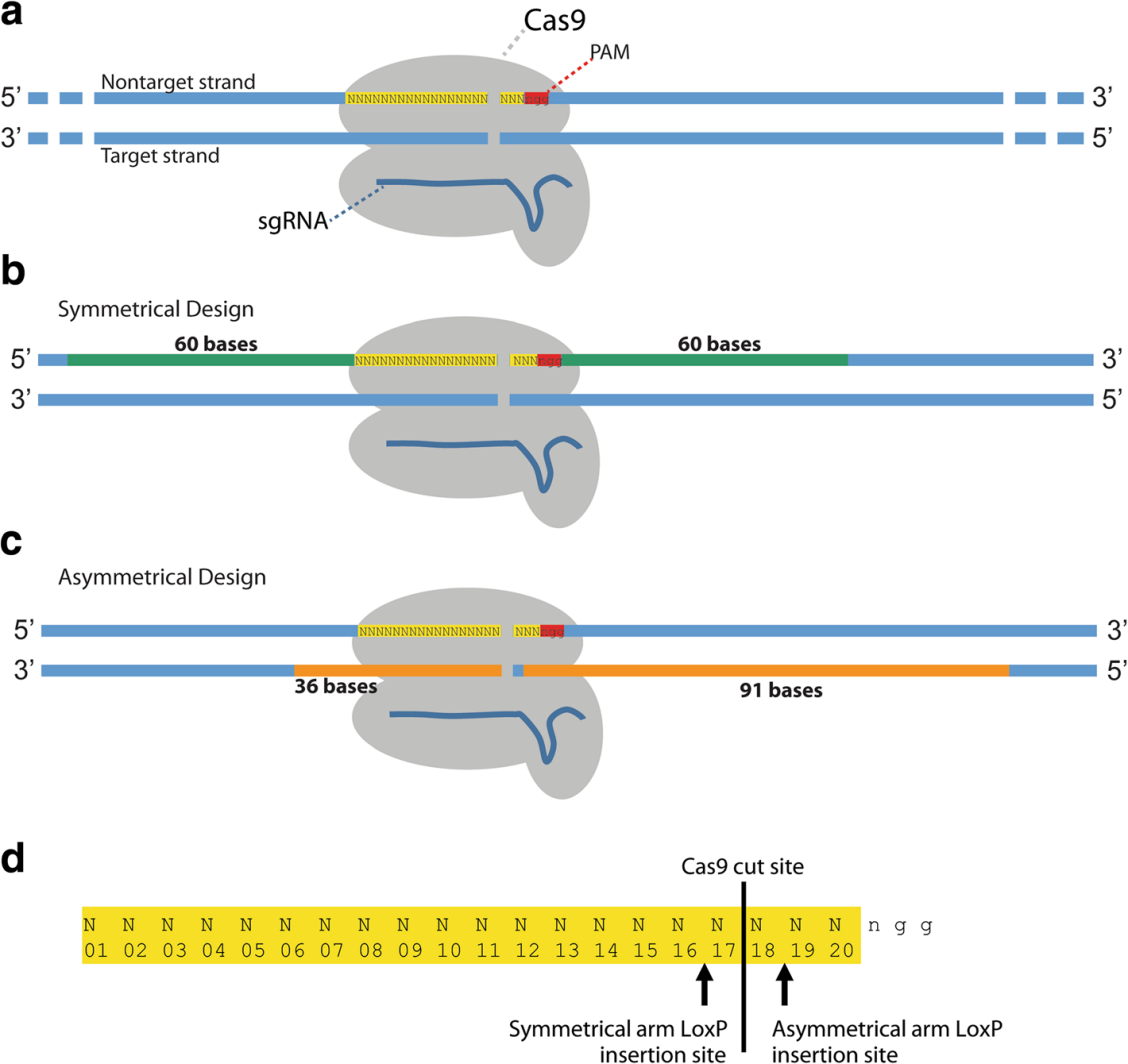
Designs for creating conditional alleles through CRISPR-mediated targeting with ssODN donor DNA. **a** Schematic for illustrating conditional targeting designs. Cas9 (gray) complexed with sgRNA (dark blue) binds to complementary DNA (blue) on the target strand after recognition of the PAM site (red). **b** Symmetrical design utilized two 60 bp homology arms, excluding the sgRNA target sequence and PAM site. The symmetrical ssODN donors were designed to be complementary to the target strand. **c** Asymmetrical design utilized a 36 bp PAM-distal and a 91 bp PAM-proximal homology arms [35]. The asymmetrical ssODN donors were designed to be complementary to the non-target strand. **d** Diagram illustrating the position of the loxP site insertion within the sgRNA target sequence in the ssODN donor. The loxP sequence was always inserted one base away from the Cas9 cut site, disrupting the sgRNA sequence in the ssODN donor and thereby preventing re-cutting by Cas9 after targeting

For approximately two-thirds of the genes targeted for conditional allele production, we utilized ssODNs harboring conventional symmetrical homology arms 60 bp in length, not including the sgRNA sequence or the PAM site (Fig. 1a, b). To prevent unwanted mutagenesis of the correctly targeted allele, we modified the target site in the ssODN donor by placing the loxP sequence, along with a restriction enzyme recognition sequence, between bases 16 and 17 of the target sequence, 1 base away from the predicted cut site of Cas9 (Fig. 1d). The sequence of the ssODN was complementary to the target strand. Richardson et al. [35] previously showed that ssODNs complementary to the non-target strand and with asymmetric homology arms (91 bp PAM-proximal, 36 bp PAM-distal) optimizes ssODN annealing to the DNA strand first released by Cas9 after DSBs are generated and increases the frequency of HDR events in vitro. Thus, the remaining third of the genes targeted for CRISPR/Cas9-generated conditional alleles were used as asymmetrical homology arms to test if this approach increases HDR efficiency in vivo (Fig. 1c). The location of the inserted loxP site was shifted within the sgRNA sequence, to be on the PAM-proximal arm, between bases 18 and 19 of the sgRNA sequence (Fig. 1d) [35].

**Fig. 2:**
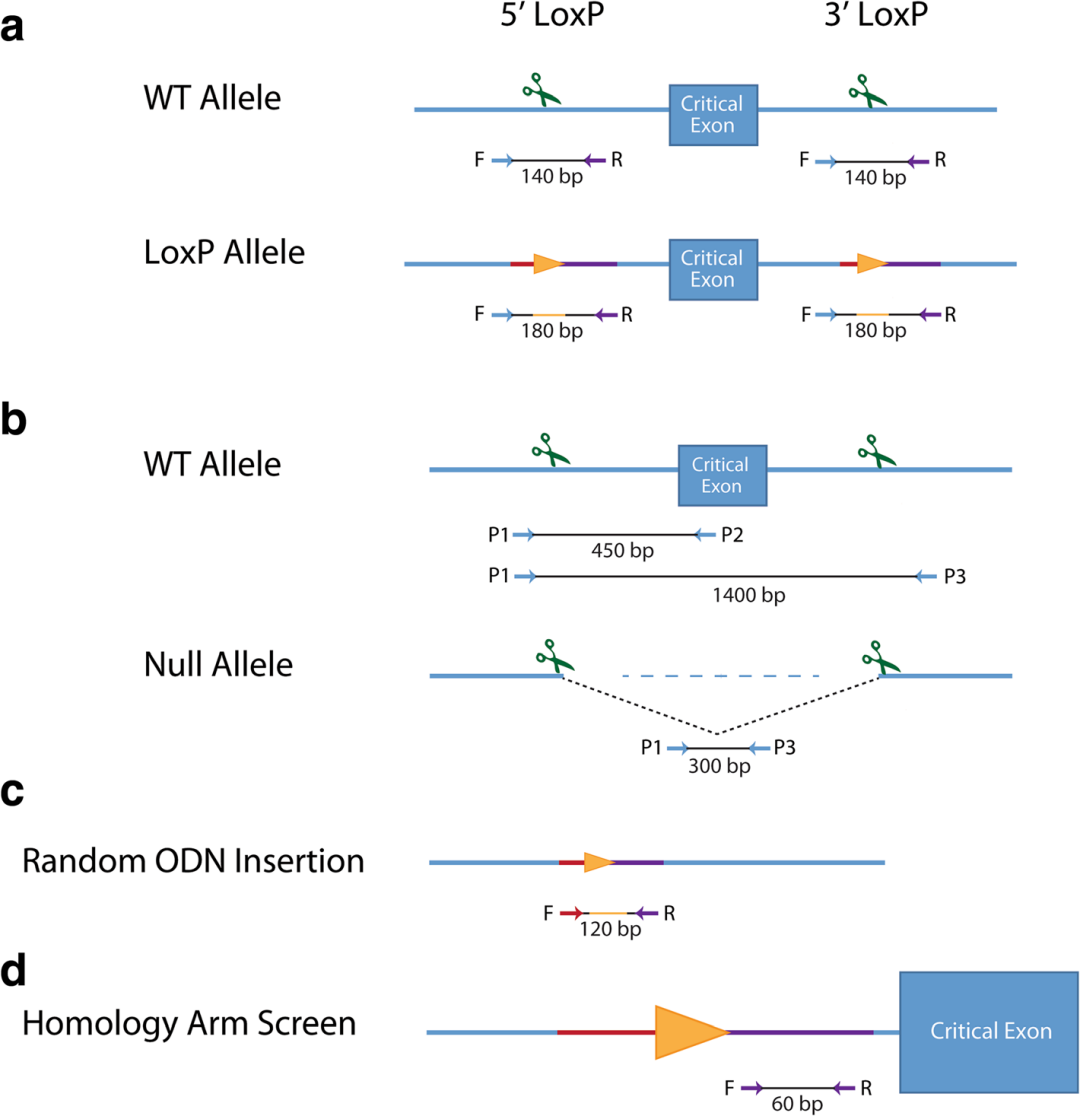
Screening strategies for HDR and NHEJ alleles, and random ODN insertion. Relative positions of the primers and approximate sizes of PCR products are listed below each allele. Scissors represent target sites. **a** Genotyping schemes for detecting loxP donor sequences. Orange triangles represent loxP sites, with representative homology sequence color coded on blue DNA strand. **b** Genotyping for NHEJ events utilizes a three-primer system, with P1 being shared between P2 and P3. Primers P1 and P3 reside between 100 to 200 bp outside of the target site (an average deletion product size is depicted). The P1 + P3 primer pair may not always amplify a wild-type product, if the target sequences are too far apart. 1400 bp represents the average distance between loxP insertion sites. **c** Random ODN insertion PCR primers reside internal to homology arm sequence, and will amplify the expected size product if the ssODN donor has been incorporated elsewhere in the genome, away from the critical exon, in addition to the on-target locus. **d** Primers for the homology arm screen were used in a SYBR-green quantitative PCR reaction from DNA samples from the N1 generation, using β-actin as a two-copy normalization control

Genotyping assays were designed to identify HDR-mediated insertion of each loxP site and NHEJ-mediated deletion of the critical exon between the two sgRNA target sites. To detect incorporation of each individual loxP site, PCR-based genotyping assays were designed to amplify at least 140 bp around the target sgRNA site for each loxP, with at least one primer outside the ssODN sequence (Fig. 2a). Successful incorporation of the loxP site was identified by a 40 bp shift in the PCR product, representing the 34 bp loxP site and a 6 bp restriction enzyme site 5′ of the loxP sequence. To detect wild-type and critical exon deletion (null) alleles generated by NHEJ resolution of the two CRISPR/Cas9-generated DSBs, a three primer PCR genotyping scheme was employed (Fig. 2b). Both loxP PCR reactions and deletion/wild-type PCR reactions were performed on all live-born (hereafter referred as F0) mice.

### CRISPR/Cas9-mediated HDR with ssODNs

To generate genome-edited mice, pronuclear stage C57BL/6NJ embryos were microinjected with Cas9 mRNA, two sgRNAs, and two ssODNs into the cytoplasm. Approximately 200 embryos were microinjected per session per gene (Additional file 2: Table S2). Litter sizes were noted for metrics of live-born pups based on the total number of zygotes injected. Microinjections and transfers resulted in fairly robust litters, with an average of 28 pups per attempt (14% of transferred embryos were live born).

**Fig. 3:**
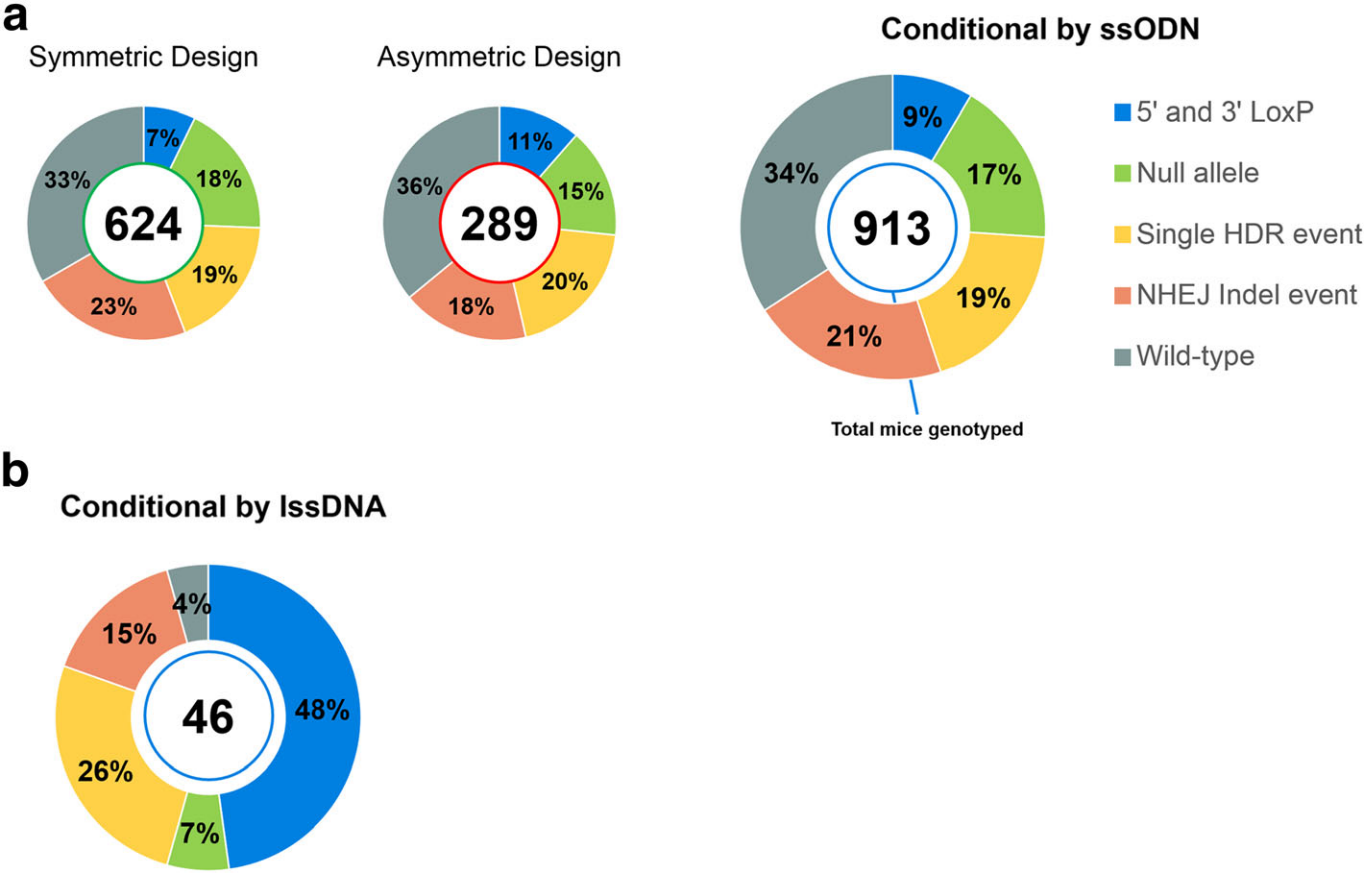
Conditional KO ssODN (**a**) and lssDNA (**b**) targeting attempts. Each donut chart represents the summation of each allele type for all F0 mice genotyped, by ODN donor. In the center of the chart is the total number of F0 mice genotyped. Percentages of each allele type of the total number of mice genotyped are listed on each segment. *5′ and 3′ loxP:* Includes animals genotyped for both 5′ and 3′ loxP sites, irrespective of the presence of any additional alleles (e.g., animals with 5′ loxP, 3′ loxP and a null allele detected); *Null allele:* Includes animals genotyped for a null allele, which may also have a single HDR and/or NHEJ indel event; *Single HDR event:* Includes animals genotyped for a single HDR event with or without additional indel events; *NHEJ Indel event:* Animals in which only indel alleles were observed. **a** Summary of genotypes identified from ssODN targeting attempts, and symmetric and asymmetric homology arm designs. **b** Summary of genotypes identified from four lssDNA targeting attempts

Of the genes targeted with symmetrical homology arm donors, 15 of 22 (68%) genes had at least one putative founder mouse with both 5′ and 3′ loxP sites integrated into the genome; 21 of 22 genes had at least one F0 mouse with a null allele (Additional file 3: Figure S1A and Additional file 2: Table S2). In total for the F0 animals from microinjections of symmetrical homology arm donors, 7% had both 5′ and 3′ loxP sites integrated and 18% harbored a null allele (Fig. 3a). Together, these results highlight the potential of generating two allele types with a single microinjection.

Because previous reports suggested that using ssODNs with asymmetric homology arms improves HDR efficiency in vitro [35], we next tested whether asymmetric homology arms increase HDR efficiency in vivo. Using the same sgRNAs, two loci (*Il1rl1* and *Eif2s2*) were targeted for conditional allele production using asymmetric and symmetric ssODN designs (Additional file 3: Figure S1B and Additional file 2: Table S2). Targeting attempts with symmetric homology arm ssODNs successfully produced founders with 2 loxP sites at *Il1rl1* but not *Eif2s2*. Asymmetric homology arm ssODN microinjections successfully produced animals with 2 loxP sites at *Eif2s2* but not *Il1rl1*. Both sets of microinjections produced founders with null alleles; therefore, failure to produce animals containing 2 loxP sites was not the result of either sgRNA failing to induce DSBs. We attempted conditional allele generation at an additional 8 loci using asymmetrical homology arm ssODNs (Additional file 3: Figure S1C), which resulted in 7 out of 8 genes with at least one putative founder mouse harboring both 5′ and 3′ loxP sites in the genome. Thus, of the microinjections attempted with asymmetric ssODNs, 8 of 10 (80%) produced a putative founder animal, which was a modest but not significant improvement over the microinjections employing symmetric ssODNs (68% of microinjections producing a putative founder, *p* = 0.68; Additional file 4: Table S3). Moreover, all asymmetric ssODN microinjections resulted in at least one F0 mouse with a null allele (Additional file 3: Figure S1B and C, and Additional file 2: Table S2). In total, 11% of F0 mice from asymmetric ssODN microinjections had both 5′ and 3′ loxP sites integrated and 15% harbored a null allele (Fig. 3a). Importantly, the distance between sgRNA target sites (range of 250 to 4500 bp) had no observable influence on the frequency of both loxP sites integrating into the genome (Additional file 5: Figure S2A).

In summary, for the 32 microinjections using pairs of ssODNs, 23 produced at least one F0 mouse with both 5′ and 3′ loxP sites integrated into the genome and 31 produced at least one F0 mouse with a null allele. Of all F0 mice, approximately 9% had 2 loxP sites integrated and 17% had a null allele (Fig. 3a). For some targeting attempts, instances of both alleles were observed in a single mouse; 24% of the putative 2 loxP founder animals were identified with both 5′ and 3′ loxP sites and a null allele (Additional file 2: Table S2).

### In *cis* versus in *trans* integration of loxP sites with ssODNs

One of the drawbacks of targeting loxP sites into the genome with paired ssODNs is that the HDR events occur independently, with the potential for targeting to occur on separate chromosomes (in *trans)*. Thus, once founders harboring both 5′ and 3′ loxP sites were identified, we next tested for co-transmission of the loxP sites to the next generation as definitive proof that loxP sites were integrated on the same chromosome (in *cis*). For 22 of the 23 lines having putative conditional null founders, breeding identified at least one founder for 18 lines with loxP sites in *cis* (81%; Table 1 and Additional file 2: Table S2). Putative conditional null allele founders from the *Eif2s2* targeting with asymmetric ssODNs were not bred. On average, 63% of bred founders were found to transmit loxP sites in *cis* and 37% in *trans* (Additional file 2: Table S2). Comparisons between targeting with symmetric or asymmetric homology arm ssODNs revealed no significant difference in the prevalence of loxP sites in *cis*, although the data trends towards symmetrical homology arms having a slight advantage of targeting in *cis* (*p* = 0.07, Additional file 4: Table S4). Interestingly, while there were few projects attempted with greater than 2.5 kb between sgRNA target sites for loxP introduction, we did observe a greater frequency of loxP in *cis* integration when genomic distances between target sites was larger than 2250 bp (Additional file 5: Figure S2B). Additional microinjections will need to be performed to test for associations between in *cis* integrations and loxP distance.

**Table 1:** Transmittance of 2 loxP sites to N1 generation

Donor	2 loxP Loci Bred	loxP in *cis*	In *cis*, NOT mutated	Overall success
2× ssODNs	22	18 (81%)^a^	14 (77%)^b^	44%^c^
Symmetric	15	14 (93%)	10 (71%)	45%
Asymmetric	7	4 (57%)	4 (100%)	40%

^a^Percentage represents number of conditional alleles with loxP in *cis* over total 2 loxP loci bred; ^b^Percentage represents number of loci with no mutations detected over all loci with loxP in *cis*. ^c^Percentage represents number of loci with no mutations detected over all loci attempted

Next, for the lines in which loxP sites were found to be in *cis*, Sanger sequencing was used to confirm the fidelity of the loxP sites in founder progeny. Of the putative founder mice selected for breeding from the 18 lines with loxP in *cis*, Sanger sequencing identified only 14 with at least one founder with both loxP sites intact (Table 1). Comparison between targeting with symmetric or asymmetric homology arm ssODNs revealed no significant difference in the predisposition for one homology arm time to integrate in *cis* without mutation (*p* = 0.52, Additional file 4: Table S5). The majority of mutated loxP sites observed by sequencing contained truncated sequences, although single-base changes were also observed (Additional file 6: Figure S3).

### HDR efficiency with ssODNs is influenced by DSB efficiency

**Fig. 4:**
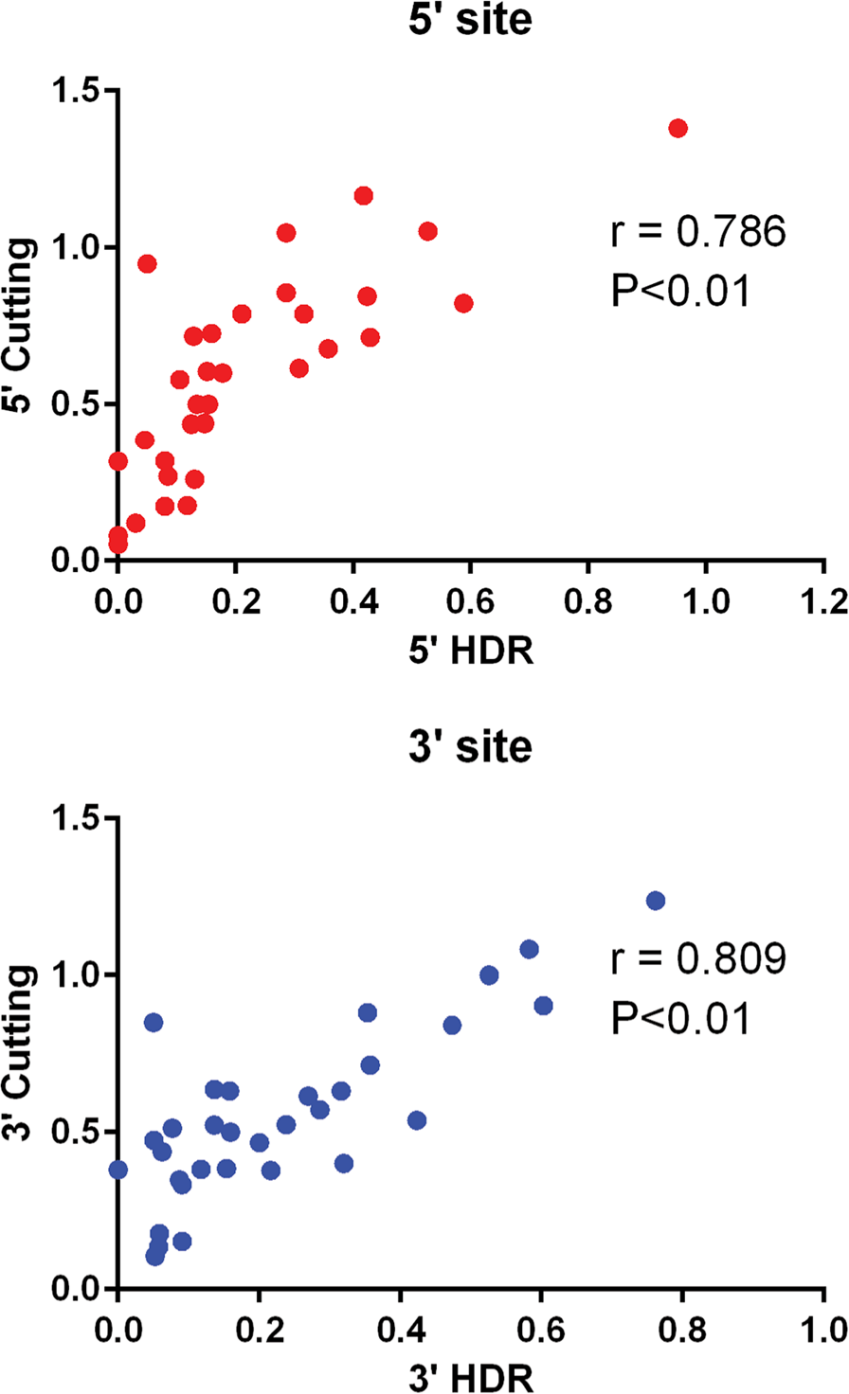
Pearson correlations for 5′ and 3′ loxP sites. Data plotted based on number of F0 mice with an HDR event (x-axis) versus any evidence for a DSB generated at the respective sgRNA site, 5′ and 3′, such as a NHEJ indel, HDR event, or the formation of a null allele (y-axis)

To determine whether HDR efficiency was influenced by the efficiency of sgRNA-guided, Cas9-mediated DSB production, we examined our loxP PCR genotyping results for PCR products indicative of NHEJ-generated indels at the sgRNA target sites (Fig. 2a). The incidence of indel events at individual sgRNA target sites, the incidence of exon deletion events, which occur when DSBs are generated at both the 5′ and 3′ sgRNA target sites, and the incidence of loxP insertion, which can only occur following a DSB event, from all F0 mice were then used to calculate the overall frequency (hereafter referred to as cutting frequency) of Cas9-generated DSBs at each target site. Cutting frequency was then compared to HDR frequency for all sgRNAs. Importantly, we detected a significant positive correlation between HDR and cutting frequency (Fig. 4). Thus, sgRNA-guided, Cas9-mediated DSB production efficiency is a critical determinant of HDR efficiency.

### Conditional allele design and genotyping schemes for CRISPR/Cas9-mediated HDR with lssDNAs

As technology development has progressed since the adoption of CRISPR/Cas9 genome editing in mammals, methods for generating long (1000–2000 base) single-stranded oligodeoxynucleotide donor molecules (lssDNAs) have been published [14, 22]. Miura et al. [14] described a novel technique of using double-stranded DNA as a template for in vitro transcription of RNA from a T7 promoter sequence, and then reverse-transcribing the RNA to generate a single-stranded cDNA molecule. Quadros et al. [22] demonstrated the feasibility of obtaining conditional and reporter alleles at several loci using lssDNA donor molecules in CRISPR-based targeting in mouse zygotes.

**Fig. 5:**
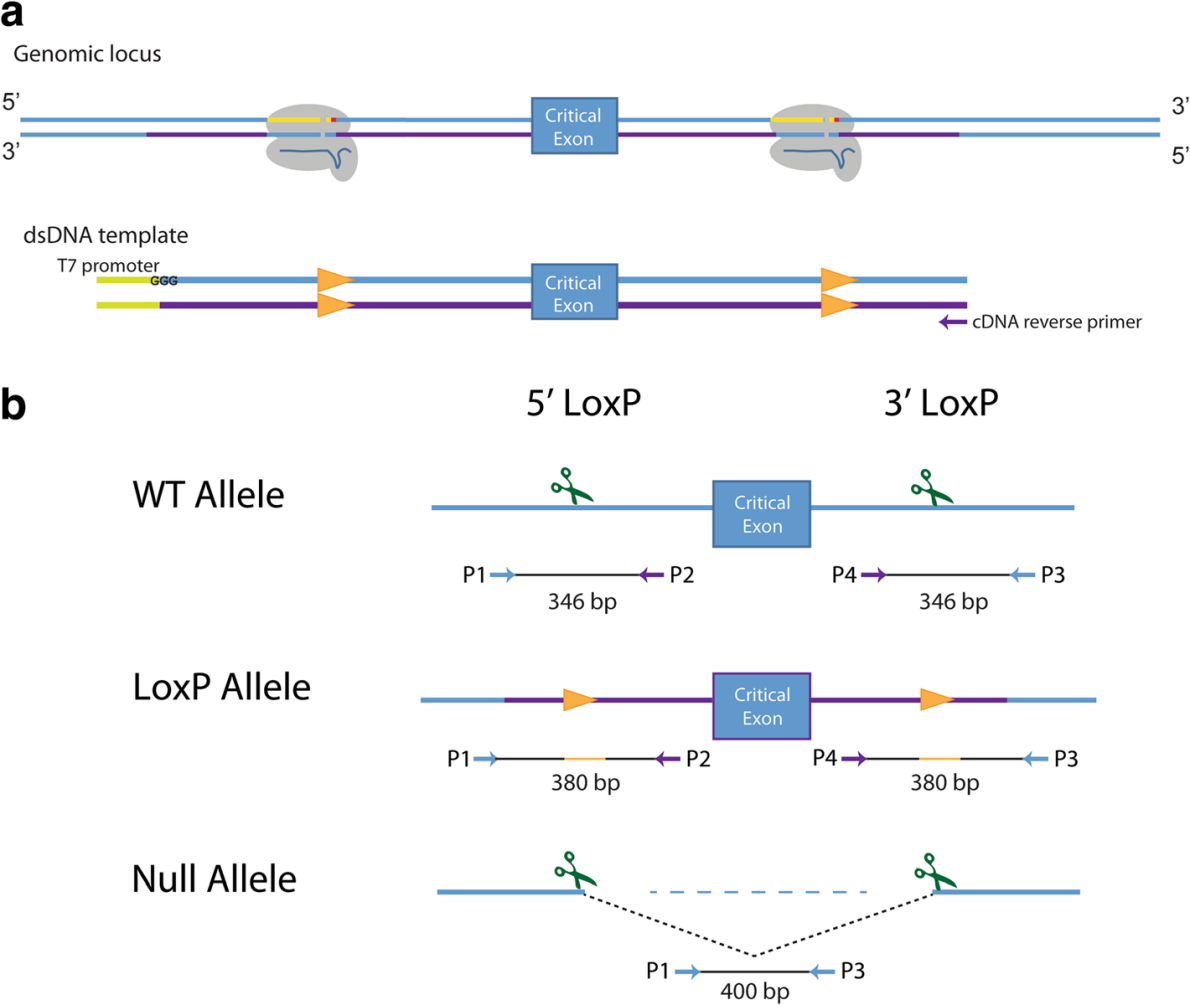
Designs for creating and genotyping conditional alleles through CRISPR-mediated targeting with lssDNA donors. **a** Schematic for illustrating lssDNA conditional targeting designs. Cas9 (gray) complexed with sgRNA (dark blue) binds to complementary DNA (blue) on the target strand after recognition of the PAM site (red). A double-stranded DNA template is purchased with 100–200 bp homology arms. The 5′ homology arm begins with a GGG and ends with the 5′ sgRNA cut site. The 3′ homology arm terminates at an appropriate cDNA primer. **b** Genotyping schemes for detecting floxed and null alleles. Orange triangles represent loxP sites, with representative homology sequence color coded on blue DNA strand. Genotyping for a null allele uses primers P1 and P3 from each loxP genotyping reaction, which can also detect a full-length wild-type product (not shown) due to the smaller distance between loxP sites than typical designs from ssODN targeting attempts

To test whether CRISPR/Cas9-mediated HDR with pairs of sgRNAs and a single lssDNA could be used to efficiently and reliably produce conditional null alleles for high-throughput production, we targeted an additional four genes for CRISPR/Cas9 genome editing. We utilized resources already available from the IMPC for each gene to select the critical exon or exons to be ‘floxed’, and the genes to be targeted with this donor type had a critical region less than 1000 bp. Pairs of sgRNAs were identified to generate DSBs within introns 5′ and 3′ to the critical exon(s) as previously described for ssODNs (Additional file 1: Table S1). For genes to be targeted for conditional alleles by lssDNA (*Eif2s2*, *Mdh2*, *Megf11*, and *Cd44*), homology arms flanking the region to be floxed varied between 100 and 200 bp in length as needed to incorporate elements for synthesis of the lssDNA [14, 22]. For the 5′ homology arm, the length was determined starting at the 3′ end, corresponding to base 17 in the 5′ sgRNA sequence (where the DSB would be initiated by Cas9), and terminating at the 5′ end with a trinucleotide stretch of guanines. A T7 promoter was added 5′ to the second guanine, ensuring that the DNA donor template sequence contained three guanines at the 3′ end of the T7 promoter. For the 3′ homology arm, an anti-sense PCR primer was selected from a 200 bp window starting at base 17 in the 3′ sgRNA sequence, to be used to initiate reverse-transcription in the cDNA synthesis (Fig. 5a).

Genotyping assays were designed to identify HDR-mediated insertion of each loxP site and NHEJ-mediated deletion of the critical exon between the two sgRNA target sites. To detect incorporation of each individual loxP site, PCR-based genotyping assays were designed to amplify approximately 350 bp around the target sgRNA site for each loxP, with one primer outside the lssDNA homology arm (Fig. 5b). Successful incorporation of the loxP site was identified by a 34 bp shift in the PCR product. To detect critical exon deletion (null) alleles, the forward 5′ loxP primer and 3′ loxP reverse primer were used to amplify a deletion product. Both loxP and deletion PCR reactions were performed on all putative founder mice.

### CRISPR/Cas9-mediated HDR with lssDNAs

To generate genome-edited mice, pronuclear stage C57BL/6NJ embryos were microinjected with Cas9 mRNA, two sgRNAs, and a single lssDNA into the cytoplasm. Approximately 200 embryos were microinjected per session per gene (Additional file 2: Table S2). Microinjections and transfers resulted in notably smaller litters than microinjections with two ssODNs, with an average of 12 pups per attempt (8% of transferred embryos were live born). Of the genes targeted with lssDNAs, all had at least one F0 mouse with both 5′ and 3′ loxP sites integrated into the genome (Additional file 3: Figure S1D). A much larger proportion of genotyped mice had both 5′ and 3′ loxP sites targeted – 48% of F0 mice from the lssDNA microinjections, compared to 9% of F0 mice from ssODN microinjections (Fig. 3). Direct comparisons for founder generation across donor types can be performed for *Eif2s2*, as the same sgRNAs were used for targeting with both symmetric and asymmetric ssODNs, as well as lssDNAs. Putative founders were identified in 0% and 9% of F0 mice with symmetric and asymmetric ssODNs, respectively, while the lssDNA donor microinjection identified 67% of F0 mice with 2 loxP sites. When comparing between ssODNs and lssDNAs for all mice genotyped, animals with null alleles occurred less frequently when using lssDNA (7% of all mice genotyped) in comparison to microinjections using pairs of ssODN (17% of all mice genotyped, Fig. 3). F0 conditional mice selected for breeding had loxP sites and floxed exons sequenced concurrently. Founder breeding from all four genes successfully transmitted the conditional allele, with both loxP in cis (Additional file 2: Table S2). However, one of the bred founders had several single base mutations in the loxP sites.

Two of the four microinjections using lssDNAs produced no animals with null alleles, which could be attributed to the loci attempted or session effects during the microinjection (Additional file 3: Figure S1D). Direct comparison of *Eif2s2* targeting across different donors produced null alleles in 16% and 18% F0 mice with symmetric and asymmetric ssODNs, respectively, while the lssDNA donor microinjection produced no mice with a null allele (Additional file 3: Figure S1B, D). A larger sample size would be needed to assess if there is a significant decrease in the ability to generate both conditional and null allele founders from a single microinjection with an lssDNA.

### Off-target mutagenesis

Although the frequency of CRISPR/Cas9 off-target mutagenesis appears to be lower in mouse zygotes compared cell lines [19, 36, 37], off-target mutagenesis is still possible, in particular for sgRNAs with off-target sites harboring one or two mismatches [36, 38]. Thus, to minimize the probability of off-target events [39], only sgRNAs predicted to have off-target sites with three mismatches or more were employed. To rule out off-target events in our conditional null allele lines, High Resolution Melt analysis of N1 animals derived from founders for seven targeted genes were screened for inheritance of off-target mutagenesis events at the top three predicted off-target sites for each sgRNA. N1 mice were selected for screening over founders due potential mosaicism in founders, which might obscure detection of an off-target mutagenesis event. We did not detect mutagenesis in any of the off-target sites screened (see Additional file 7: Table S6 for off-target locations).

### Random insertion of DNA donors and reintegration of excised genomic intervals

The potential also exists for donors to randomly integrate into the genome. To screen for random ODN insertion (ROI), genotyping screens with primers internal to the donor ssODN or lssDNA were designed to be run on all F0 mice from a subset of microinjections (Fig. 2c and Table 2). The results of this genotyping were compared to the initial genotyping performed to detect the targeting of a loxP site, which uses a design with at least one primer outside a homology arm and that can detect only an on-target allele (Fig. 2a). Therefore, if an F0 animal had a loxP site detected by the ROI screen, but not the on-target loxP genotyping screen, we consider this a random insertion event. F0 mice from six ssODN and two lssDNA microinjections were screened for ROI events. Of the ssODN microinjections, five out of six had at least one mouse with an ROI event (Table 2), with an observed incidence of 3–18% of F0 mice affected. Both of the assayed lssDNA donor targeting attempts identified animals with a ROI event, with an observed incidence of 20–30% of F0 mice affected. Therefore, random integration of single-stranded DNA donors does occur.

**Table 2:** F0 screening for random ODN insertion

Gene	Donor design	5′ ROI events	3′ ROI events	Total F0 s	ROI %
Il1rl1	ssODN – symmetric	0	0	19	0%
Abat	ssODN – symmetric	3	0	19	16%
Slc2a12	ssODN – symmetric	1	1	12	17%
Sugct	ssODN – symmetric	0	1	33	3%
Kctd7	ssODN – symmetric	1	4	28	18%
Uqcr10	ssODN – asymmetric	0	1	34	3%
Eif2s2	lssDNA	0	2	9	22%
Cd44	lssDNA	1	2	10	30%

**Fig. 6:**
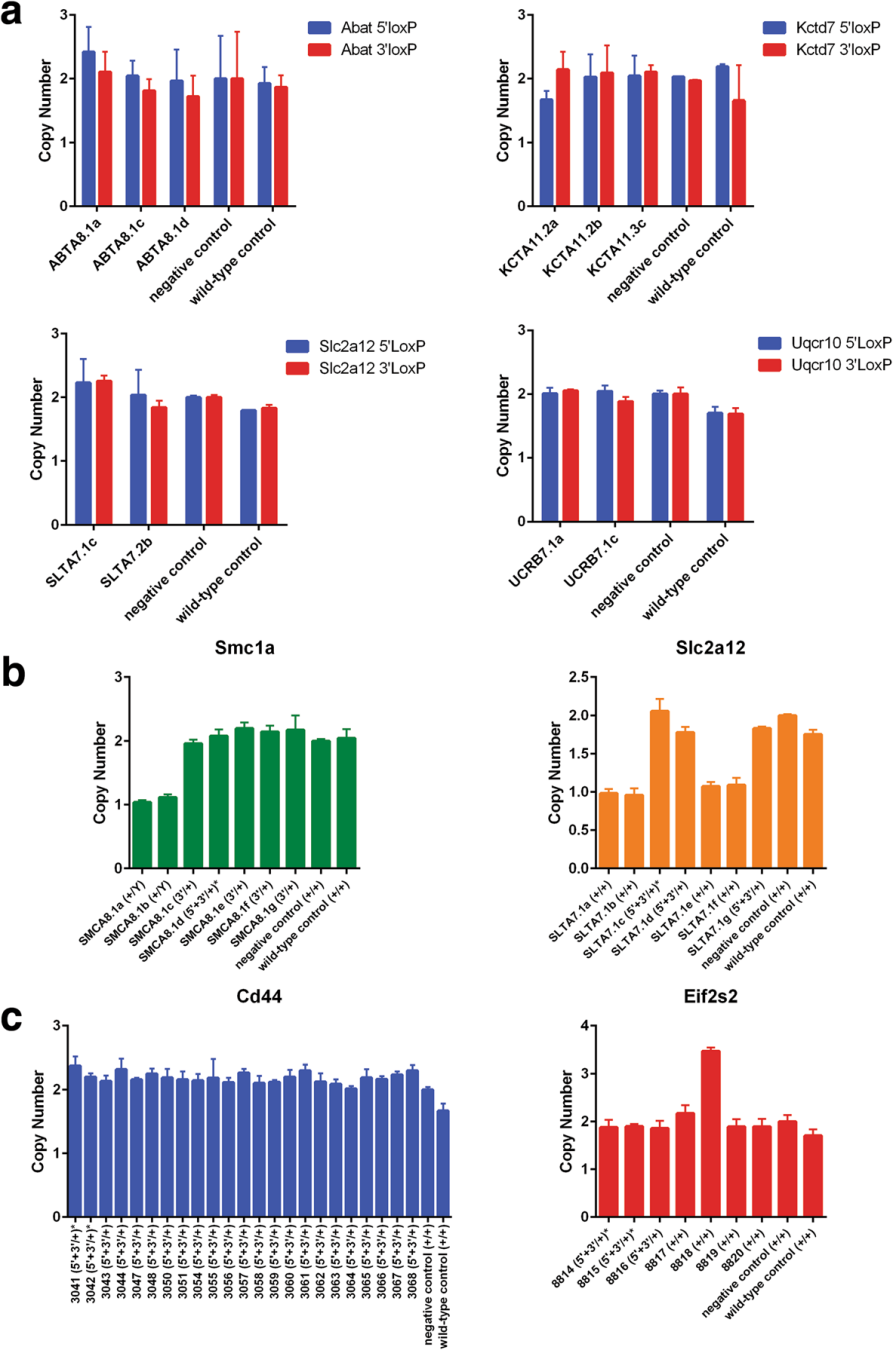
**a** Copy number data from quantitative SYBR Green PCR assays for *Abat*, *Kctd7*, *Slc2a12*, and *Uqcr10*, using primers following the design introduced in Fig. 2d for both 5′ and 3′ loxP ssODN donors. All mice screened for each conditional null attempt were heterozygous conditional null mice from the N1 generation that were sequence confirmed for the conditional allele. Negative control samples were CRISPR-targeted N1 mice that were wild-type for the allele being analyzed. **b, c** Copy number data from TaqMan^®^ Copy Number assays for (**b**) *Slc2a12* and *Smc1a* (paired ssODN conditional null targeting attempts) and (**c**) *Eif2s2* and *Cd44* (single lssDNA conditional null targeting attempts) using N1 progeny from a single founder. Genotypes are listed with the mouse ID. Animals with an asterisk to the right of the genotype were sequence-confirmed for the conditional null allele. Of note, *Smc1a* is located on the X chromosome, thereby males only have one copy of the gene. As in (**a**), negative control samples were CRISPR-targeted N1 mice that were wild-type for the allele being analyzed

Based on our assay method, on-target integration obscures detection of ROI in putative founder animals. Therefore, we used alternative assays based on sequence copy number to detect inheritance of ROI events from putative founders in N1 animals. For ssODNs, N1 animals derived from putative conditional null alleles for *Abat*, *Kctd7*, *Slc2a12*, and *Uqcr10*, and harboring both loxP sites, were subjected to Quantitative SYBR Green real-time PCR using primers that amplify a portion of the homology arm (Fig. 2d). Conditional N1 offspring should have a homology arm copy number of 2. Any N1 animal with more than two copies would be considered to have inherited both an on-target and ROI allele. No increase in copy number was observed in the N1 animals screened (Fig. 6a). Therefore, while our screen of F0 animals suggests that ROI occurs in animals lacking an on-target HDR event, the failure to detect ROI in N1 animals derived from founders in which on-target HDR occurred suggests that the likelihood of both a ROI and HDR event happening in the same animal is low.

A recent publication revealed that genomic intervals excised using pairs of sgRNAs can reintegrate into the target locus during NHEJ repair, resulting in inversions or duplications [40]. This finding also raises the possibility that excised genomic intervals could randomly integrate into other sites in the genome. Therefore, we used TaqMan copy number assays employing probes specific to critical exon sequences to test whether N1 progeny (wild-type and heterozygous for conditional null alleles) inherited an extra copy of the critical exon. For ssODNs, N1 animals from a single founder for *Smc1a* and *Scl2a12* were assayed (Fig. 6b). None were found to harbor an extra copy of the critical exon. However, we did identify N1 progeny, previously genotyped as wild-type, with a single copy for *Slc2a12*. These mice likely harbor an exon deletion allele that resulted in large flanking sequence deletions that removed the primer site(s) used for the null allele PCR reaction (Fig. 2b).

We used similar TaqMan assays to assess critical exon copy number in N1 progeny from *Cd44* and *Eif2s2* conditional null allele founders generated with lssDNAs. However, when using lssDNAs, it is more challenging to discriminate between random insertion of the lssDNA and duplications from the excised DNA. Either would cause an increase in exon copy number. We did not detect an increase in copy number for any N1 progeny derived from the *Cd44* conditional allele founder; however, we did detect a single N1 mouse from the *Eif2s2* founder that had one extra copy of the critical exon in the genome (Fig. 6c). Importantly, the N1 animal with the extra copy did not inherit the on-target conditional null allele. Therefore, to determine if the extra copy was due to ROI or reintegration (duplication) of the excised genomic interval, we used our ROI screen (Fig. 2c) to test whether loxP sites could be detected. Mice genotyped positive for both loxP sites (data not shown), indicating that the increase in copy number was due to a ROI that was inherited from the founder. Together, these results reveal that it is critical to perform sufficient allele quality control checks to ensure that selected founder or N1 mice with desired on-target alleles do not harbor any additional unwanted DNA insertions, which may affect the overall phenotype of the desired mouse model.

## Discussion

The effective generation of conditional alleles through the use of CRISPR/Cas9 has historically been challenging due to the inefficiencies of HDR in mouse zygotes. Plasmid donors have a relatively high cost and long lead time in production, as well as low frequency of targeting [6, 8], which renders their use unsuitable for high-throughput production. Studies have attempted to shorten homology arms in plasmids from existing targeting vector libraries for their use as donors with CRISPR/Cas9 genome editing [18]. However, any cost savings achieved through the use of existing plasmids is quickly lost in time needed to modify the donors prior to their use with CRISPR/Cas9. In comparison, ssODNs have proven to be simple to design, relatively cheap to produce, and an efficient donor molecule for CRISPR/Cas9-mediated HDR in mouse zygotes. While ssODN donors have been widely used to introduce sequence alterations at single sites (e.g., point mutations and epitope tags) [6–8, 41], their use for conditional allele production (two sites of HDR at the same locus) has been more limited in scale [8, 13, 19–21]. More recently, lssDNAs have proven to be an effective donor for CRISPR/Cas9-mediated insertion of floxed exons into the genome of mouse zygotes [22]. In the present study, we have tested the use of CRISPR/Cas9-mediated HDR with ssODN pairs and single lssDNAs to generate conditional null allele mice for high-throughput production. Based on our results and those of others who have employed ssODNs and lssDNAs [14, 22], we propose that lssDNAs are the best donor template for reliable and efficient scale production of mouse lines harboring conditional alleles.

### Conditional allele production efficiency with ssODNs and lssDNAs

When we compare conditional allele production efficiency between the two different single-stranded DNA donor types, lssDNAs were substantially more efficient than ssODNs at producing F0 mice harboring intact and in *cis* loxP sites (48% vs. 9%, respectively). The requirement for two independent HDR events when using ssODNs versus one HDR event when using lssDNAs significantly contributed to differences in conditional allele production efficiency. Using pairs of sgRNAs and ssODNs, both sgRNAs need to efficiently facilitate Cas9-generated DSB production if HDR is to insert both loxP sites into the genome. Our data (Fig. 4) clearly demonstrates that HDR efficiency is directly affected by the efficiency of DSB production. Thus, if one Cas9-sgRNA complex is suboptimal at generating a DSB, the rate of conditional allele production will be significantly reduced. If time and resources are permitting, pre-screening for Cas9-sgRNA complexes that are highly efficient at generating DSBs and designing ssODNs around those target sites would most likely increase the probability of targeting both loxP sites into the genome of the same mouse. Although two sgRNAs were employed with each single lssDNAs to induce sequence replacement by HDR, it is possible that only one Cas9-generated DSB at only one of the two sgRNA target sites was needed to facilitate HDR, thereby increasing overall HDR efficiency with lssDNAs.

Comparisons between the microinjection conditions presented in this study, the adjoining publication by Codner et al., and other publications using ssODN or lssDNA donors [22, 42, 43], highlight the possibility of achieving allele targeting with a variety of concentrations and microinjection routes (i.e., pronuclear or cytoplasmic). Concentration differences between our microinjection attempts with ssODNs and lssDNAs exemplify the flexibility of the CRISPR/Cas9 system. Cas9 mRNA, sgRNA, and donor DNA concentrations were lower in our lssDNA microinjections compared to our ssODN microinjections (see Methods), but HDR efficiency was not adversely affected. Thus, we propose that there is not one correct condition to be employed for single-stranded donor DNA microinjections. Each core or laboratory must optimize their conditions, starting from conditions known to work, to produce optimal results.

A second obstacle for conditional allele production with ssODNs is achieving two independent HDR events on the same allele. However, of the 35 putative conditional allele founders resulting from paired ssODN injections and backcrossed to generate N1 mice, 22 (63%) loxP sites transmitted in *cis* and 13 (37%) transmitted in *trans*, revealing a possible preference of HDR events occurring on the same allele. Furthermore, when distance between sgRNA target sites was considered as a factor, in *cis* integration occurred more frequently compared to in *trans* integration (89% vs. 11% of bred founders, respectively) when the target sites were greater than 2250 bp apart. Thus, employing pairs of ssODNs for conditional allele production might be an efficient approach for loci that require loxP integration at distances over 2250 bp. Importantly, of the putative conditional null founders that were produced using lssDNA donors and were subsequently bred, all transmitted both loxP sites in *cis*. Thus, in *cis* integration of loxP sites is more reliable with lssDNAs.

A recent study described a practice of generating conditional allele mice through microinjection or electroporation of one sgRNA and ssODN to target one loxP site into the genome at the one-cell embryo stage, followed by a subsequent microinjection or electroporation of a second sgRNA and ssODN to target the second loxP site into the genome of the same embryos at the two-cell stage [44]. This approach decreased the potential of generating undesired interval deletion alleles in favor of the desired HDR (conditional) allele. Of the limited loci that the authors attempted, they observed decreased survival rates of zygotes translating into fewer live born animals, in addition to targeting rates marginally better than that reported herein using paired ssODNs as donors in a single microinjection. Our high-throughput production to create conditional alleles would not be able to sustain the added costs or efforts required to target loxP sites sequentially.

### Null allele production when using ssODNs and lssDNAs

From an IMPC resource standpoint, it is highly advantageous to produce an exon deletion (null) allele and a conditional allele F0’s from the same microinjection. Thus, conditional allele mice for distribution to the scientific community and null allele animals for the phenotyping pipeline can be generated at the same time. Importantly, of the 913 F0 mice resulting from paired ssODN microinjections, 18% harbored a null allele. Furthermore, of the 9% of F0 mice harboring a putative conditional allele, 24% also harbored a null allele (Additional file 2: Table S2). Animals that showed evidence of both NHEJ and HDR events were especially valuable if there was no evidence of the wild-type allele in the genotyping. It could be inferred that separate targeting events had occurred on opposite alleles and that the loxP sequences had been targeted in *cis*.

Importantly, as the distance between sgRNA target sites increased, the number of mice harboring null alleles decreased, in particular when the distance between target sites exceeded 2250 bp (Additional file 5: Figure S2A). It is likely that NHEJ repairs the two DSBs generated by the two sgRNAs independently as the distance between the target sites increases, resulting in two small indels rather than an interval deletion.

Importantly, our primer design for identifying null alleles (Fig. 2b) limits our ability to detect NHEJ repair events that result in larger deletions (100–200 bp) of sequences flanking the DSB points. Such deletions remove primer sequence(s) for null allele genotyping (Fig. 2b). As evident from our TaqMan assay, large flanking sequence deletions do occur (Fig. 6b). However, for our null allele production, we prefer a smaller ‘scar’ from the resulting NHEJ repair to minimize the potential of affecting splicing of neighboring exons that may be affected by large deletions of flanking sequence.

Not surprisingly, the increase HDR efficiency observed when using lssDNAs resulted in a decrease in F0 mice harboring a null allele (7%). In fact, only two of the four lssDNA injections generated F0’s with a null allele. Thus, although lssDNAs are more efficient at producing conditional allele founders, this comes at the expense of null allele founder generation.

When estimating the number of F0 mice needed to screen to achieve both a putative conditional null allele founder and a null allele founder from the same microinjection, the rate limiting allele differs between donor DNA types. For paired ssODN attempts, generating the conditional null allele is rate limiting, as we observed 9% of genotyped mice having both 5′ and 3′ loxP sites (Fig. 3a) and 63% of bred putative conditional null founders harbored their loxP sites in *cis* (Additional file 2: Table S2). Therefore, for every 18 F0 mice born from a conditional null targeting attempt with paired ssODN donors, 1 (5.7%) of the F0 s should have both loxP sites targeted in *cis*, while 3 (17%) F0 mice should be null founders. Of course, there is a high variability of targeting efficiency locus to locus, so additional mice may have to be screened. For lssDNA attempts, the null allele is rate limiting, as we observed only 7% of genotyped F0 mice harboring a null allele. Thus, for every 15 F0 mice born from a conditional null targeting attempt with a single lssDNA donor, 1 (7%) of the F0 s should be a null founder, while 7 (48%) F0 mice should have both loxP sites targeted in *cis*.

### Mutagenized sequences at sites of HDR

Unfortunately, we did observe a proportion of successfully targeted loxP sites with mutagenesis when using paired ssODNs, which together with in *trans* integration, substantially reduced the efficiency of conditional allele founders. Two theories have been postulated to account for mutations observed. One hypothesis accounts for microhomology between a region of the donor DNA and the endogenous allele [45], which may cause indel mutations due to microhomology-mediated end joining. Alternatively, synthesis errors when producing the ssODNs may become apparent as single copies are integrated into genomic DNA. A recent publication targeting loxP sites in the *Dock7* locus sequence confirmed 1–2 bp deletions and a 1 bp substitution within the loxP site and/or surrounding sequence arising from incorrectly synthesized donor DNA [19]. Sequencing of N1 mice revealed single base deletions, which might be more attributable to synthesis errors, but large 10–12 bp deletions (Additional file 6: Figure S3) seem to suggest microhomology-mediated end joining. Using lssDNAs, we also observed a founder with a successfully targeted floxed exon but with single base mutations within both loxP sites. This alteration can most likely be attributed to a synthesis errors in the gBlock DNA template or acquisition of an error during the in vitro transcription or the cDNA synthesis steps of ssODN production. Thus, when using either ssODNs or lssDNAs as DNA templates for conditional allele production, it is imperative to sequence across the region of HDR.

Importantly, although introduction of sequence errors in loxP sites (ssODNs or lssDNAs) or floxed exons (lssDNAs) are fatal flaws, errors in intronic sequences flanking loxP sites or exons can be tolerated if the base change(s) are predicted to not alter splicing or gene expression. Although altered intronic sequences are undesirable in the age of precision genome engineering, they have in the past been tolerated and proven to be mostly inconsequential when producing conditional null allele mice using traditional ES cell-based gene targeting. For example, ES cell clones harboring tm1a knockout first alleles and used by IMPC/KOMP^2^ to generate and phenotype thousands of knockout mouse lines have intronic microdeletions at sites where loxP sequences were introduced in the targeting vectors [25].

### Off-target mutagenesis screening

Due to the high throughput nature of our KOMP^2^ production pipeline, N1 offspring, rather than founder animals, were screened for off-target mutagenesis. Founders are often mosaic, which can result in tissues processed for DNA extraction and screening harboring no cells in which an off-target event occurred or too few cells for off-target events to be reliably detected. Thus, even if off-target mutagenesis is not detected in the founder, we would still screen N1 progeny to verify results from the founders. Moreover, due to mosaicism, the off-target allele may not be transmitted through the germline. Importantly, in the event that a bred founder harbored an off-target event, it would likely segregate from the on-target allele in N1 animals. When possible, we increased the probability of on and off-target mutation segregation a priori by attempting to select sgRNAs for which the top five predicted off-target sites are not on the same chromosome as the target allele. Thus, the probability of an off-target mutagenesis event segregating with the targeted allele is low.

### Random insertion of DNAs

Data presented in this study highlights the potential for both ssODNs and lssDNAs to randomly integrate in F0 mice, as revealed by a standard PCR assay using primers internal to the ssDNA donor sequence (Table 2). This ROI assay is limited in that it cannot distinguish between on-target integration and random donor insertions in founder animals. Statistically, the occurrence of ROI and on-target HDR, two independent events, should be rare. For ssODNs, of the 145 F0 animals analyzed for ROI, only 12 (8%) were found to have a ROI event (Table 2). Only 9% of F0 animals targeted with ssODNs harbored both a 5′ and 3′ loxP site (Fig. 3). Thus, only 0.72% of F0 s (8% × 9%) would be predicted to harbor a ROI and a putative conditional allele. To determine the frequency of random donor insertions inherited in the N1 generation, we performed additional copy number assays in sequence-confirmed conditional null allele heterozygous mice, and detected no increases in copy number for either ssODN for the lines screened.

Conversely, if a random insertion event occurred in a putative conditional null founder, there is a high probability that these two events are not genetically linked, and would segregate independently in the N1 generation. The companion publication by Codner et al. [43] did observe founders with increased copy number, in at least one conditional targeting attempt, as measured by ddPCR and TaqMan reagents. This founder was subsequently bred and the progeny were screened for copy number. N1 animals with and without the desired conditional null allele had additional copies of the screened exon. Therefore, the authors concluded that the random integration event was not physically linked to the desired targeted allele in the founder [43]. They also suggested that there are limited ways to determine whether the increased copy number was due to random insertion of the donor DNA or a duplication event from the excised DNA created from CRISPR/Cas9-induced DSBs.

In our study, putative conditional null founders were bred and the N1 progeny was screened using qPCR and TaqMan reagents to identify an increased copy number of the critical exon. We did not detect increases in copy number in any of the sequence-confirmed heterozygous conditional null N1 animals. A single previously genotyped wild-type N1 progeny from a putative conditional null founder was identified with increased copy number (*Eif2s2*; Fig. 6c). We were able to perform additional PCR assays to determine that the increased copy number was likely to be a ROI event inherited from the founder. Therefore, while excised genomic DNA intervals can lead to DNA duplications [40] and screening steps should be considered for quality assurance of newly generated mouse lines, ROI is likely to be more frequent and of greater concern for quality assurance. Future publications could explore the potential for random insertion of DNAs to integrate at sites of off-target mutagenesis induced by CRISPR/Cas9. Similar to random integration of transgenes, a ROI event could alter gene expression or function at the site of integration, and produce phenotypes not associated with the on-target modifications. Importantly, as long as ROI occurs on a separate chromosome from the on-target locus, breeding can be used to segregate the two alleles.

### ssODN homology arm symmetry

ssODN arm symmetry had varying effects on HDR efficiency, the rate of in *cis* integration, and mutagenesis at the site of HDR. Overall success, defined as projects with one founder harboring in *cis* loxP integration without sequence errors, was similar for symmetric and asymmetric ssODNs (45% vs. 40% respectively, Table 1), but the success rates appear to be driven by different factors. Success at producing at least one putative founder with in *cis* integration of loxP sites was better for symmetric than asymmetric ssODNs (93% vs. 57% of projects, respectively) while success at producing a putative founder with unaltered in *cis* loxP sites was better for asymmetric than symmetric ssODNs (100% vs. 71% of projects with an in *cis* founder, respectively). These observed differences could be due to the loci selected for targeting. *Il1rl1* and *Eif2s2* were targeted using both symmetric and asymmetric ssODNs, but had opposite results, wherein symmetric ssODNs performed better for *Il1rl1* and asymmetric ssODNs worked better for *Eif2s2* (Additional file 3: Figure S1B). However, aspects of the designs may have some influence on outcomes, in particular loxP mutagenesis. The asymmetric ssODN design employed [35] used sequences complementary to the non-target strand and introduced novel sequences (i.e., loxP) on the PAM proximal side of the Cas9 cut site (Fig. 1d). As previously reported, distal homology arms of asymmetric ssODNs appear to initiate strand invasion during synthesis-dependent strand annealing-mediated HDR [35]. Thus, loxP sequences in asymmetric ssODN might have been protected from potential mismatches with genomic DNA during repair. In comparison, loxP sites were introduced on the PAM distal side of the Cas9 cut site of target sequences when using symmetric ssODNs. Additional studies testing loci in parallel with each donor design would be necessary to verify that our observed differences in in *cis* integration and loxP mutagenesis are due to differences in homology arm symmetry and loxP placement relative to the Cas9 cut site.

### Considerations for using lssDNAs

Importantly, although lssDNAs harboring floxed exon sequences appear to be more efficient than paired ssODNs harboring loxP sites at producing conditional alleles, there are several limiting factors to lssDNAs that must be considered. From a null allele production standpoint, the decreased efficiency of generating both a putative conditional null founder and a null founder from a single microinjection is interesting. The higher rate of HDR targeting with lssDNAs compared to loxP targeting with ssODNs may be a cost–benefit decision to be made when attempting to obtain two alleles from a single microinjection. Of course, obtaining a conditional null allele can lend to the production of a null allele with Cre recombinase-mediated recombination. Another interesting point of the lssDNA microinjections were the generally smaller litter sizes from roughly the same number of embryos microinjected. The synthesis of the lssDNA may produce unwanted contaminants that are toxic to embryos. Generally, the smaller litter sizes are not of significant concern given the high targeting efficiency of the lssDNAs to generate the desired allele.

In order to generate lssDNAs via *in vitro* transcription and cDNA synthesis, a double-stranded DNA template is required. If the critical region exceeds the limits of synthetic DNA blocks currently commercially available, or if the sequence is unable to be synthesized as a double-stranded DNA template due to its complexity or repetitive nature, plasmid-cloned DNA can be used as a template. However, the use of plasmids will increase time and cost of production. Furthermore, in vitro transcription and reverse transcription are error prone and can introduce unwanted mutations in loxP sequences or exon sequences during lssDNA production. There are other synthesis options available, including exonucleases to preferentially degrade a single strand of DNA [46], use of a biotin-labelled primer [47], or nicking enzymes to be used with plasmid DNA to separate a single strand [45]. All of these methods also have limitations. The in vitro transcription/cDNA synthesis method, exonuclease digest, and biotin-labelled primers require the use of a dsDNA template. Amplification of the dsDNA template can also introduce errors even when using high-fidelity polymerase. Nicking enzymes require plasmid synthesis and gel purification for the liberated lssDNA. There is also a consideration for the length of the single-stranded DNA donor, since longer sequences (> 5 kb) may have a tendency to break and be targeted independently at each DSB site. Importantly, the recent availability of synthesized lssDNAs (Megamers, IDT DNA) [22] may address sequence error issues with lssDNA production, but limitations on sequence length (up to 2000 bp) and synthesis of complex/repetitive sequences can still be problematic. With the available current methodologies for lssDNA production, the use of paired ssODNs as DNA templates for CRISPR/Cas9-mediated HDR may be the only option for generating conditional alleles at some loci.

## Conclusions

CRISPR/Cas9-mediated HDR with lssDNAs is currently the most efficient option for large scale production of mice harboring conditional null alleles. However, ssODNs are a viable alternative for conditional allele production when an lssDNA donor is not feasible. Importantly, regardless of the type of single-stranded donor employed, it is of upmost importance to screen new mouse models generated by CRISPR/Cas9-mediated HDR with single-stranded DNA donors for errors at the site of repair and random integration of the donor sequences into the genome. While our efforts to date have focused on approaches for scaled production of mice harboring conditional null allele for IMPC/KOMP^2^, we will continue to test methods for generating larger and more complex lssDNAs for scaled production of mice harboring knock-in or flexible alleles using CRISPR/Cas9 genome editing.

## Methods

### Mouse strains

C57BL/6NJ and ICR mice were purchased from the Jackson Laboratory (Bar Harbor, ME) and maintained in the AAALAC-accredited animal facility at Baylor College of Medicine. All studies were reviewed and approved by the Institutional Animal Care and Use Committee of BCM and in accordance with National Institutes of Health guidelines for the Care and Use of Laboratory Animals.

### Conditional allele design

Critical exon(s) to be floxed were identified using the targeting vector designs already created for each gene by the IMPC [34]. sgRNAs were selected using either the crispr.mit.edu or Wellcome Trust Sanger Institute (WTSI) Genome Editing websites; sgRNAs chosen had no fewer than three mismatches and were at least 100 bp proximal to the exon to be floxed and at least 100 bp from any neighboring exons. All sgRNA and off-target information can be found in links provided in Additional file 1: Table S1 and Additional file 7: Table S6, respectively. Symmetrical ssODN design used homology arms 60 bp in length, not including the sgRNA sequence or the PAM site. The loxP sequence was introduced between bases 16 and 17 of the sgRNA target site (predicted cut site of Cas9). The ssODN sequence was complementary to the target strand (Fig. 1b). Asymmetrical ssODN design was attempted for 10 of the 30 genes targeted, which used asymmetric homology arms (91 bp PAM-proximal, 36 bp PAM-distal) and a ssODN sequence complementary to the non-target strand (Fig. 1c). Novel restriction sites for *Xho*I, *Eco*RI, or *Bam*HI were placed immediately 5′ of the loxP integration site. LssDNA design selected sgRNAs at least 50 bp 5′ or 3′ of the critical exons to be floxed. LoxP sites were introduced between bases 18 and 19 of the sgRNA target site and no restriction sites were included. T7 promoters were added to 5′ homology arms, at least 100 bp 5′ to the sgRNA site and 5′ to a trinucleotide stretch of guanines. The 3′ homology arm length was at least 100 bp, and terminated with a suitable primer for cDNA synthesis (Fig. 5a). Donor sequences are listed in Additional file 1: Table S1; for the lssDNA donors, the T7 promoter, loxP sites, and critical exon(s) are in uppercase, all other sequence in lowercase.

### ssODN donors

For ssODN donors, custom Ultramer^®^ DNA oligonucleotides were purchased from Integrated DNA Technologies (Coralville, IA). Synthesis of lssDNA donors was initiated with a DNA template obtained from Integrated DNA Technologies (gBlock) (Fig. 5a). The DNA template was synthesized in vitro with mMESSAGE mMACHINE T7 Ultra transcription kit (ThermoFisher, cat. # AM1345) and purified using MEGAclear kit (ThermoFisher, cat. # AM1908) after DNase treatment, as described previously [14, 48]. The cDNAs were reverse transcribed from synthesized RNAs using SuperScriptIV Reverse Transcriptase (ThermoFisher, cat. # 18091050) using gene-specific primers (Additional file 1: Table S1), followed by RNase treatment. Unincorporated nucleotides and enzymes were removed from the cDNA by the Qiagen PCR purification kit (Cat. 28,104) and the concentrations were measured using a NanoDrop™ 2000.

### sgRNA and Cas9 mRNA preparation

sgRNAs were synthesized using DNA templates for in vitro transcription. DNA templates were produced using overlapping DNA oligonucleotides in a high-fidelity PCR reaction [49]. The PCR products were first purified using the QIAQuick PCR purification kit and used as a template for in vitro transcription of the sgRNA with the MEGAshort script T7 kit (ThermoFisher, AM1354). Following in vitro transcription, RNA was purified using the MEGAclear Transcription Clean-Up Kit (ThermoFisher AM1908). All samples were analyzed by Nanodrop to determine concentration and visualized using the Qiaxcel Advanced System using the RNA QC V2.0 kit to check the quality of RNA product before storage at −80 °C. Cas9 mRNA was purchased from ThermoFisher (A25640). All sgRNAs were reanalyzed by Nanodrop prior to assembling the microinjection mixtures. Conditional allele attempts using ssODN donors consisted of Cas9 mRNA (100 ng/μL), sgRNA (20 ng/μL, each), and two ssODNs (100 ng/μL, each) in a final volume of 60 μL of 1×PBS (RNAse-free). Conditional allele attempts using lssDNA donors consisted of Cas9 mRNA (50 ng/μL), sgRNA (10 ng/μL, each), and one lssDNA (50 ng/μL) in a final volume of 60 μL of 1×PBS (RNAse-free). Sequences of donor templates used for loxP insertion are listed in Additional file 1: Table S1.

### Microinjection of CRISPR/Cas9 reagents

C57BL/6NJ female mice, 24–32 days old, were injected with 5 IU/mouse of pregnant mare serum gonadotropin, and followed 46.5 h later with 5 IU/mouse of human chorionic gonadotropin. The females were then mated to C57BL/6NJ males, and fertilized oocytes were collected at 0.5 dpc. The BCM Genetically Engineered Mouse Core microinjected the sgRNA/Cas9/ssODN mixture into the cytoplasm of at least 200 pronuclear stage zygotes per gene attempted. Injected zygotes were transferred into pseudopregnant ICR females on the afternoon of the injection, at approximately 25–32 zygotes per recipient female.

### Genotyping

Genomic DNA was isolated from ear punches from 2-week-old pups using NaOH digestion followed by neutralization with Tris-HCl and dilution with nuclease-free water, as previously described [50]. Genomic DNA was isolated from tail clips of juvenile mice by proteinase K digestion, followed by isopropanol precipitation and resuspension in 1× Tris-EDTA. DNA was amplified by standard PCR using AmpliTaq Gold™ Fast PCR Master Mix (ThermoFisher, 4,390,939). To verify insertion of the loxP sites, primers were designed to PCR amplify a 140–180 bp region around the sgRNA site, ensuring that at least one primer resided outside the homology arms of the ssODN (Fig. 2a). To detect wild-type and null alleles generated by NHEJ, a three primer scheme was designed. Two primers bind outside the two sgRNA sites to PCR amplify deletion products, residing between 100 and 200 bp outside of the target site (an average deletion product size is depicted in Fig. 2b). A third primer was designed to reside within the predicted deleted interval, to PCR amplify a product from the endogenous, wild-type allele (Fig. 2b). All PCR products were visualized using the Qiaxcel Advanced System. Primer sequences used for genotyping are available upon request.

### DNA sequencing of cloned loxP sites in mice generated by CRISPR/Cas9

Genomic DNA was amplified as described above to visualize loxP insertions. The PCR products were cloned into competent cells using the pGEM Vector System according to the manufacturer’s protocol (Promega, A3600). Clones were screened by PCR for sequences containing a loxP site, and DNA Sanger sequenced by GENEWIZ (South Plainfield, NJ) for intact loxP sequences. Conditional alleles generated in founders from lssDNA-targeted genes were sequenced by straight Sanger sequencing of purified PCR products generated from amplifying sequences around loxP sites and critical exon(s), and traces were aligned with SnapGene (GSL Biotech LLC) or deconvoluted using Sequencher (Gene Codes Corporation).

### Statistics

Statistics for calculating significance of loxP transmission, loxP in *cis*, and loxP in *cis* not mutated were performed by Fisher’s Exact Test.

### Analysis of off-target Cas9 activity

The top three potential off-target sites for each sgRNA in the conditional targeting of *Il1rl1* (symmetric design targeting), *Abat*, *Kctd7*, *Slc2a12*, *Smc1a*, *Uqcr10*, and *Eif2s2* (lssDNA targeting) were identified using the WTSI Genome Editing website. Flanking PCR primers designed to amplify 80–180 bp amplicons are listed in Additional file 7: Table S6 with the WGE ID, location, sequence, and number of mismatches from the original sgRNA. Off-target mutagenesis was assessed by High Resolution MeltAnalysis using MeltDoctor HRM Master Mix (ThermoFisher, 4,415,440) on the QuantStudio 7 Flex Real-Time PCR System. At least three wild-type samples were analyzed concurrently with the test samples. For any sample with a HRM analysis result deviating from the wild-type sample, suggesting a mutagenesis event, PCR products were amplified with secondary sequencing primers and straight Sanger sequenced as described above.

### Analysis of random insertion of DNA donors and duplicated DNA

Genomic DNA was prepared from the F0 generation and standard PCR was performed using primers that flank the loxP sequence and resided within the homology arms of the ssODNs (Fig. 2c). The results of the internal PCR genotyping were compared to the PCR genotyping used for on-target loxP insertion. To identify random insertions of ssODNs in lines established from founder animals harboring an on-target allele, primers were designed to PCR amplify sequences within the 5′ or 3′ homology arm of the ssODN for copy number counting in N1 animals (Fig. 2d). As described previously [51], DNA samples from ssODN-targeted N1 animals and wild-type C57BL/6NJ samples were amplified with the homology arm primers and *Actb* primers with Power SYBR™ Green PCR Master Mix (ThermoFisher, 4,367,659) for qPCR to serve as a copy number internal normalizing control. Homology arm copy number was determined relative to wild-type controls. DNA samples from ssODN-targeted N1 animals, negative control (non-targeted mice), and wild-type control samples were analyzed by TaqMan^®^ Copy Number assays for *Slc2a12* and *Smc1a* (ThermoFisher, Mm000591693_cn and Mm000629524_cn, respectively; Reference gene Tfrc 4,458,367). DNA samples from lssDNA-targeted N1 animals and wild-type control samples were analyzed by TaqMan^®^ Copy Number assays for *Eif2s2* and *Cd44* (ThermoFisher, Mm00054773_cn and Mm00049884_cn, respectively; Reference gene Tfrc 4,458,367).

## Additional files

Additional file 1: **Table S1.** Design information for all genes targeted, separated by length and homology arm design. Web links are provided to NCBI and Ensembl for the annotation of each gene and exon or exons targeted, and separate web links are provided to the WTSI Genome Editing website for the selected sgRNA information. Donor sequences for each gene targeted are also listed. (PDF 115 kb)

Additional file 2: **Table S2.** Microinjection information for all 32 paired, ssODN donor and the 4 lssDNA donor microinjections, including injection concentrations for Cas9, sgRNAs (each), and donor DNAs. Column headings: Embryo, represents the number of embryos injected for each targeting attempt; Recip, the number of recipient moms utilized per targeting attempt; Tfx, the number of surviving embryos transferred into recipient females; F0 born, the number of F0 pups born for each targeting attempt; PE, percent efficiency – the number of F0 pups born divided by the number of embryos transferred; Genotyped, the number of F0 pups that survived to genotyping at 2 weeks of age; Wild-type, the number of F0 pups genotyped without any evidence of genome editing; NHEJ event, the number of F0 pups genotyped in which only indel alleles were observed; Single HDR, the number of F0 pups genotyped with a single HDR event with or without additional indel events; Null Allele, the number of F0 pups genotyped with a null allele, which may also have a single HDR and/or NHEJ indel event; 5 + 3 + N, the number of F0 pups genotyped with both 5′ and 3′ loxP sites and a null allele; 2 loxP, the number of F0 pups genotyped with both 5′ and 3′ loxP sites, irrespective of the presence of any additional alleles. Data for the breeding of 2 LoxP founders is presented in beige shading; N1 cis, the number of 2 LoxP founders that transmitted in *cis*; N1 trans, the number of 2 LoxP founders that transmitted in *trans*; Not bred, the number of 2 LoxP founders not bred. (PDF 235 kb)

Additional file 3: **Figure S1. A–D.** Individual conditional KO symmetric and asymmetric ssODN and lssDNA targeting attempts. (A) Symmetric homology arm design attempts for the 20 genes attempted. (B) Paired comparison of the symmetric and asymmetric homology arm design attempts for *Il1rl1* and *Eif2s2*. The red circle circumscribing the total number of mice genotyped indicates an asymmetric design attempt. (C) Asymmetric homology arm design attempts for an additional eight genes. (D) lssDNA-mediated attempts for four genes, which included *Eif2s2. (PDF 309 kb)*

Additional file 4: **Table S3.** Fisher’s Exact Test calculation to determine significance of obtaining a founder vs. no founder between symmetric and asymmetric homology arm designs for targeting loxP sites. **Table S4.** Fisher’s Exact Test calculation to determine significance of obtaining loxP sites in *cis* vs. loxP sites in *trans* between symmetric and asymmetric homology arm designs for targeting loxP sites. **Table S5.** Fisher’s Exact Test calculation to determine significance of obtaining loxP sites in *cis*, non-mutated vs. loxP sites in *cis*, mutated between symmetric and asymmetric homology arm designs for targeting loxP sites. (XLSX 35 kb)

Additional file 5: **Figure S2. A, B.** Frequency distributions of (A) projects with conditional, null, or both allele types, and (B) projects with loxP in *cis*, in *trans*, or both, binned by loxP distance. Project results derived from genotyping information gathered from F0 (A) and N1 (B) mice. LoxP distance calculated from genomic distance between Cas9 cut site of each sgRNA. X-axis labels indicate the median value of each bin. (A) Left Y-axis depicts the number of animals screened for each loxP distance bin, while the right Y-axis depicts the allele percentage within each loxP distance bin. (B) Left Y-axis depicts the number of founders bred for each loxP distance bin, while the right Y-axis depicts the transmission percentage for all projects within each loxP distance bin. (XLSX 254 kb)

Additional file 6: **Figure S3. A, B.** Examples of mutagenized loxP sites identified upon TA cloning PCR products from loxP PCR reactions and Sanger sequencing resulting clones from founder mice. (A) Example of truncated loxP site, and missing sgRNA target sequence in HDR founder from *Mbd2* 5’ loxP site targeting. (B) Examples of base changes and deletions in three different founders from *Il1rl1* 5’ loxP site targeting. Endogenous sequence in blue, sgRNA target site (split) in black underline, *Bam*HI sequence in gold, loxP sequence in green, PAM site in red underline. (B) Copy number data from TaqMan^®^ Copy Number assays for *Slc2a12* and *Smc1a* (paired ssODN conditional null targeting attempts) and *Eif2s2* and *Cd44* (single lssDNA conditional null targeting attempts) using N1 progeny from a single founder (PDF 63 kb)

Additional file 7: **Table S6.** Location, sequence, mismatch number, and primer sequences for off-target sites listed by gene. When necessary, sequencing primer sequences are also listed for off-target sites. (XLSX 16 kb)
