# Supplementary figures and images for: Comparative analysis of single-stranded DNA donors to generate conditional null mouse alleles
Source: BMC Biol. 2018 Jun 21;16:69. doi: 10.1186/s12915-018-0529-0 (PMC6011517; doi:10.1186/s12915-018-0529-0)

Figure S1

A.

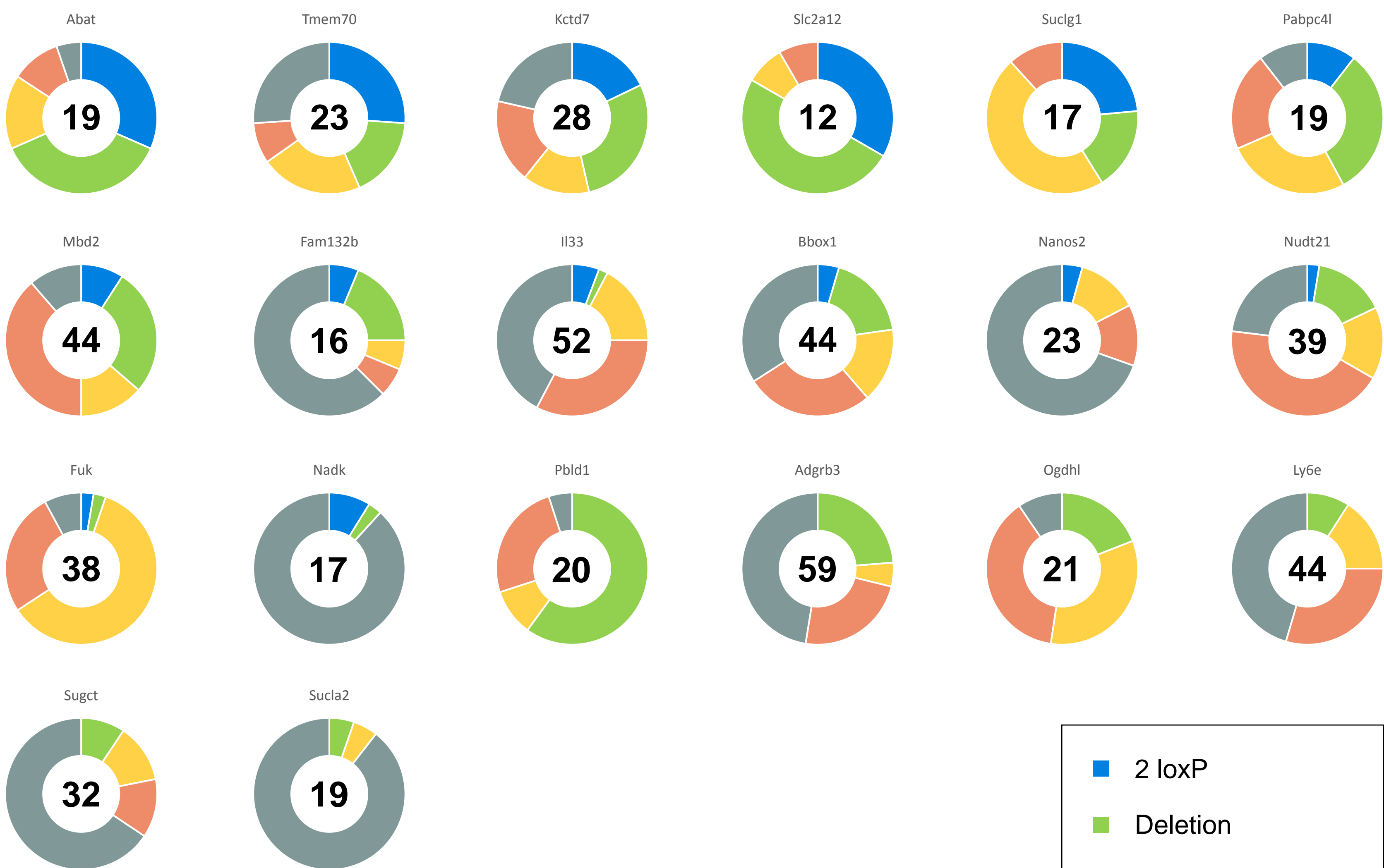

B.

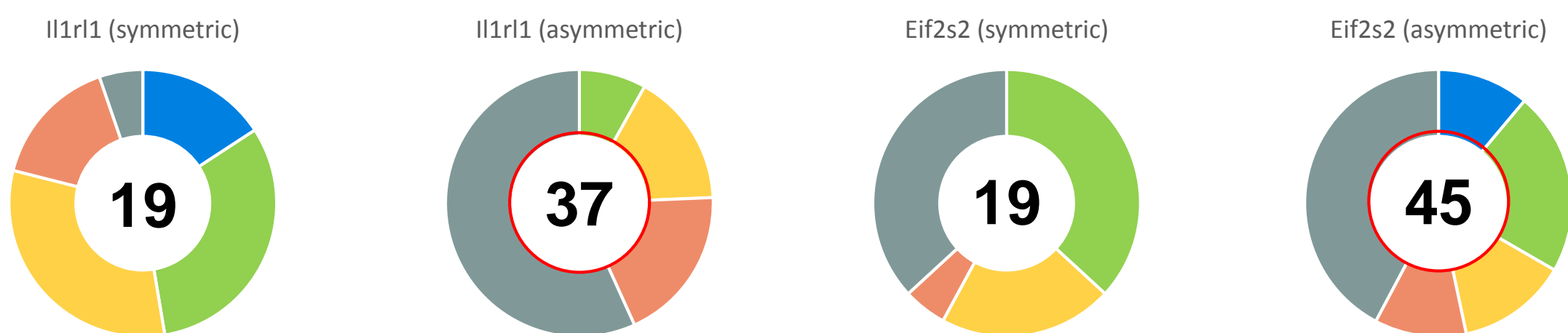

C.

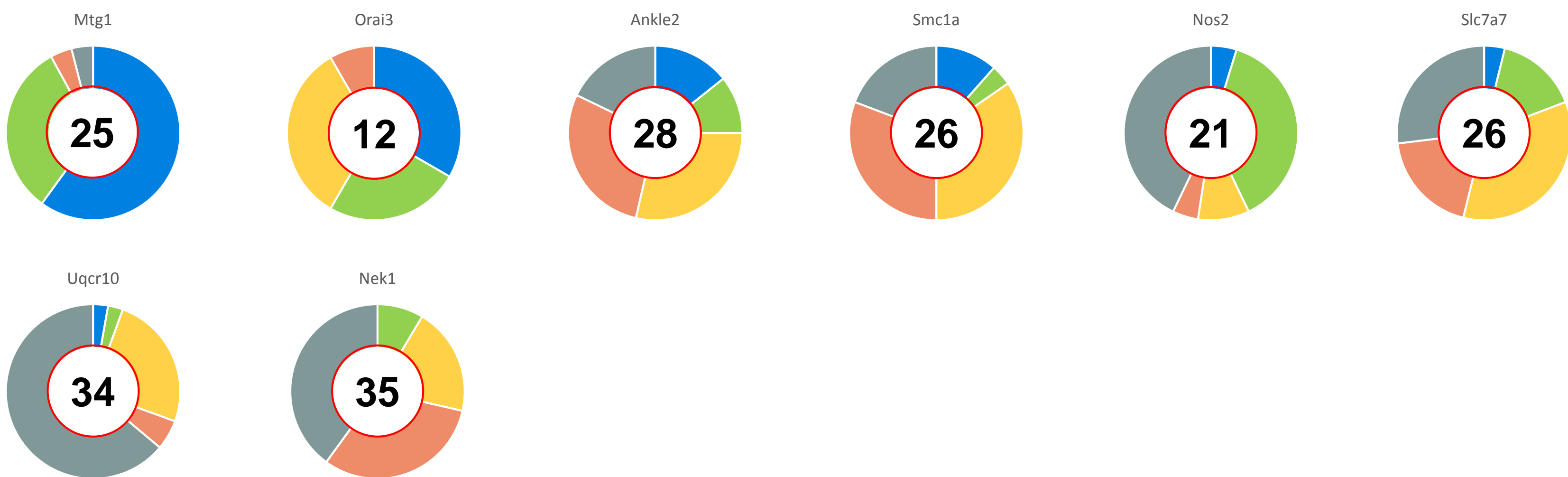

D.

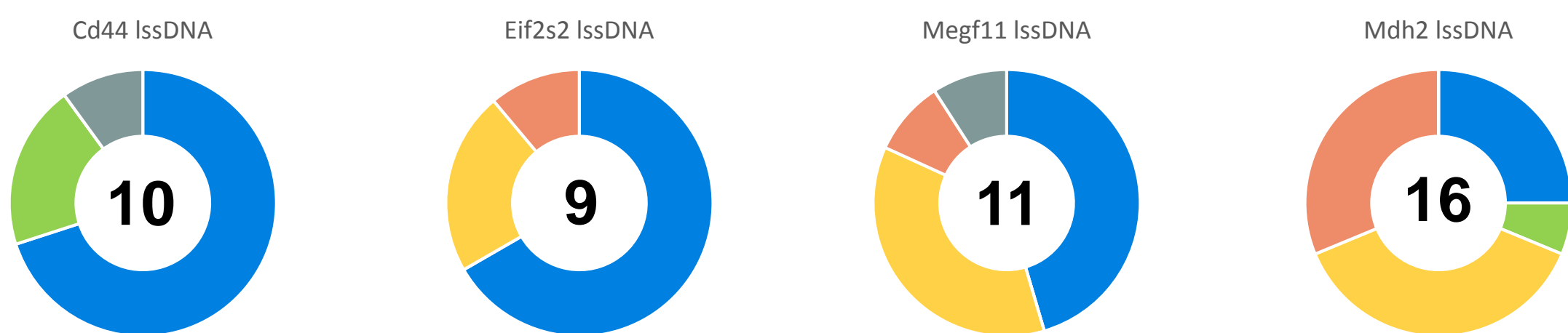

Supplement: Supplementary file 1 — Table S1. Design information for all genes targeted, separated by length and homology arm design. Web links are provided to NCBI and Ensembl for the annotation of each gene and exon or exons targeted, and separate web links are provided to the WTSI Genome Editing website for the selected sgRNA information. Donor sequences for each gene targeted are also listed. (PDF 115 kb) [file 12915_2018_529_MOESM1_ESM.pdf]

# Figure S2

A.

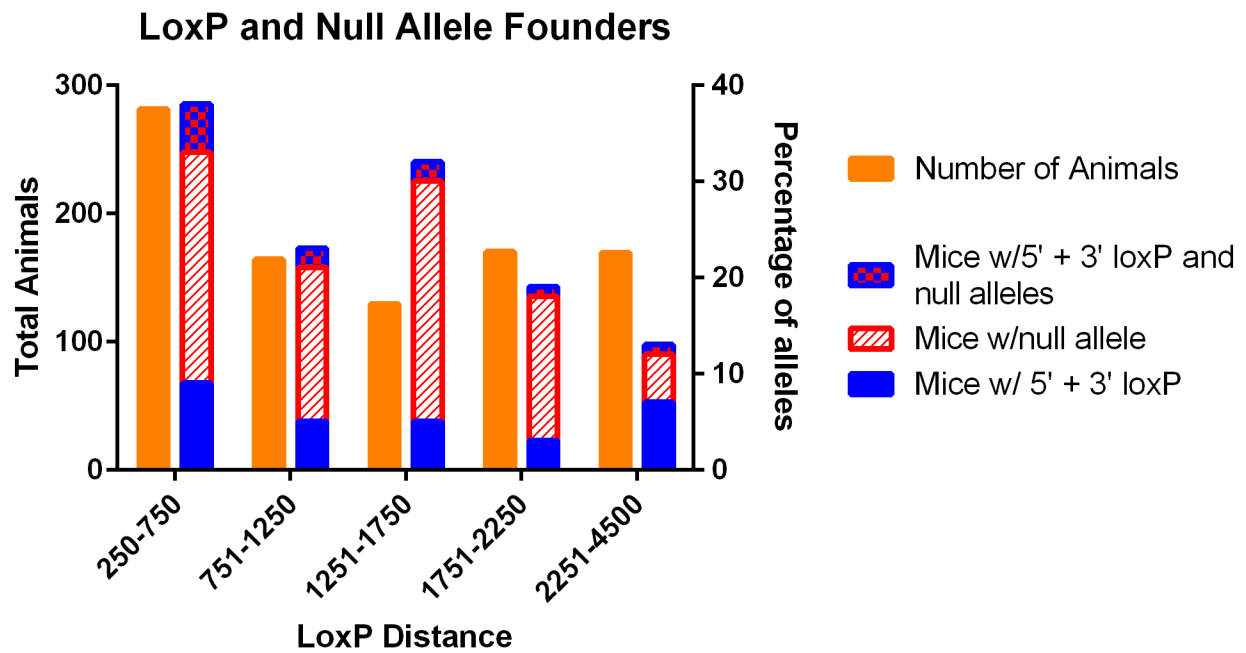

B.

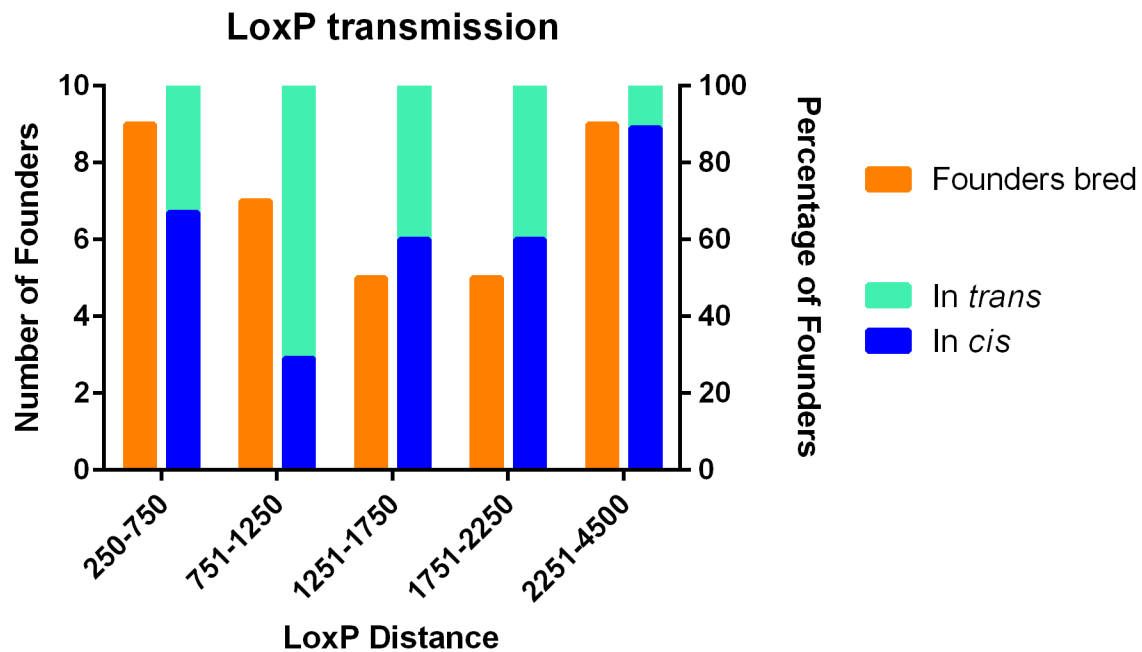

Supplement: Supplementary file 2 — Table S2. Microinjection information for all 32 paired, ssODN donor and the 4 lssDNA donor microinjections, including injection concentrations for Cas9, sgRNAs (each), and donor DNAs. Column headings: Embryo, represents the number of embryos injected for each targeting attempt; Recip, the number of recipient moms utilized per targeting attempt; Tfx, the number of surviving embryos transferred into recipient females; F0 born, the number of F0 pups born for each targeting attempt; PE, percent efficiency – the number of F0 pups born divided by the number of embryos transferred; Genotyped, the number of F0 pups that survived to genotyping at 2 weeks of age; Wild-type, the number of F0 pups genotyped without any evidence of genome editing; NHEJ event, the number of F0 pups genotyped in which only indel alleles were observed; Single HDR, the number of F0 pups genotyped with a single HDR event with or without additional indel events; Null Allele, the number of F0 pups genotyped with a null allele, which may also have a single HDR and/or NHEJ indel event; 5 + 3 + N, the number of F0 pups genotyped with both 5′ and 3′ loxP sites and a null allele; 2 loxP, the number of F0 pups genotyped with both 5′ and 3′ loxP sites, irrespective of the presence of any additional alleles. Data for the breeding of 2 LoxP founders is presented in beige shading; N1 cis, the number of 2 LoxP founders that transmitted in cis; N1 trans, the number of 2 LoxP founders that transmitted in trans; Not bred, the number of 2 LoxP founders not bred. (PDF 235 kb) [file 12915_2018_529_MOESM2_ESM.pdf]
