# Supplementary material for: Comparative analysis of single-stranded DNA donors to generate conditional null mouse alleles
Source: BMC Biol. 2018 Jun 21;16:69. doi: 10.1186/s12915-018-0529-0 (PMC6011517; doi:10.1186/s12915-018-0529-0)
Supplement: Supplementary file 3 — Figure S1. A–D. Individual conditional KO symmetric and asymmetric ssODN and lssDNA targeting attempts. (A) Symmetric homology arm design attempts for the 20 genes attempted. (B) Paired comparison of the symmetric and asymmetric homology arm design attempts for Il1rl1 and Eif2s2. The red circle circumscribing the total number of mice genotyped indicates an asymmetric design attempt. (C) Asymmetric homology arm design attempts for an additional eight genes. (D) lssDNA-mediated attempts for four genes, which included Eif2s2. (PDF 309 kb) [file 12915_2018_529_MOESM3_ESM.pdf]

Figure S3

A.

5’ loxP Site in *Mbd2* putative founder:

ssODN donor sequence

sgRNA BamHI loxP sgRNA

```
AGTTCTCAGCAGTGAGCTGTGTGTGCAGTAGCAGCATGCGCAGCACGGATCCATAACTTCGTATAGCATACATTATACGAAGTTATTTTCCGGTGTTAAG

AGTTCTCAGCAGTGAGCTGTGTGTGCAGTA-----------------------------------GCATACATTATACGAAGTTATTTTCCGGTGTTAAG
```

Genomic loxP site sequence

B.

5’ loxP Site in *Il1rl1* putative founders:

ssODN donor sequence

sgRNA BamHI loxP sgRNA

```
CATTAGAACAATGAGTAATTGCTCGATGCAGTAATTGCTCGATGCGGATCCATAACTTCGTATAGCATACATTATACGAAGTTATTCTCTGGGATTAAG

CATTAGAACAATGAGTAATTGCTCGATGCAGTAATTGCTCGATGCGGATCCATAACTTCATATAGCATACATTATACGAAGTTATTCTCTGGGATTAAG
CATTAGAACAATGAGTAATTGCTCGATGCAGTAATTGCTCGATGCGGATCCATAACTTCGTATA-C-TACATTATACGAAGTTATTCTCTGGGATTAAG
CATTAGAACAATGAGTAATTGCTCGATGCAGTAATTGCTCGATGCGGA-CCATAACTTCGTATAGCATACATTATACGAAGTTATTCTCTGGGATTAAG
```

Genomic loxP site sequences: 3 different founders
