# Supplementary material for: Comparative analysis of single-stranded DNA donors to generate conditional null mouse alleles
Source: BMC Biol. 2018 Jun 21;16:69. doi: 10.1186/s12915-018-0529-0 (PMC6011517; doi:10.1186/s12915-018-0529-0)
Supplement: Supplementary file 6 — Figure S3. A, B. Examples of mutagenized loxP sites identified upon TA cloning PCR products from loxP PCR reactions and Sanger sequencing resulting clones from founder mice. (A) Example of truncated loxP site, and missing sgRNA target sequence in HDR founder from Mbd2 5’ loxP site targeting. (B) Examples of base changes and deletions in three different founders from Il1rl1 5’ loxP site targeting. Endogenous sequence in blue, sgRNA target site (split) in black underline, BamHI sequence in gold, loxP sequence in green, PAM site in red underline. (B) Copy number data from TaqMan® Copy Number assays for Slc2a12 and Smc1a (paired ssODN conditional null targeting attempts) and Eif2s2 and Cd44 (single lssDNA conditional null targeting attempts) using N1 progeny from a single founder (PDF 63 kb) [file 12915_2018_529_MOESM6_ESM.pdf]

Tables S3-5. Fisher's Exact Test calculations for LoxP transmission differences by homology arm design

Table S3. 2 loxP site founder

	founder	no founder	total
Symmetric	15	7	22
Asymmetric	8	2	10
			p = 0.68

Table S4. LoxP in cis

	cis	trans	total
Symmetric	14	1	15
Asymmetric	4	3	7
			p = 0.07

Table S5. LoxP in cis, not mutated

	non-mutated	mutated	total
Symmetric	10	4	14
Asymmetric	4	0	4
			p = 0.52
